# Stability and adaptability assessment of red onion genotypes using AMMI, GGE, BLUP, and multivariate indices

**DOI:** 10.3389/fpls.2025.1694946

**Published:** 2025-10-22

**Authors:** Amar Jeet Gupta, Kavya V. Aribenchi, Ashwini Benke, Supriya Kaldate, Pushpa Hulagannavar, Rajiv Kale, Vijay Mahajan

**Affiliations:** Department of Crop Improvement, -Indian Council of Agricultural Research (ICAR) -Directorate of Onion and Garlic Research, Pune, Maharashtra, India

**Keywords:** onion, stability, GEI, GGE biplot, AMMI biplot, MGIDI, GYT, BLUP

## Abstract

**Introduction:**

Red onion (*Allium cepa* L.) productivity and quality are highly contingent on environmental context, necessitating rigorous genotype evaluation across sites and seasons.

**Methods:**

Twenty-four red onion genotypes were evaluated in multi-environment trials (METs) across fixed, agro ecologically diverse Indian locations, during two consecutive rabi seasons (2023-24). As the same locations were used in both years, they were treated jointly as eight distinct environments (E1-E8) to quantify genotype × environment interaction (GEI) and identify broadly or specifically adapted cultivars. An integrated analytical pipeline combined GGE and AMMI biplots with mixed-model BLUPs, including the Harmonic Mean of the Relative Performance of Genotypic Values (HMRPGV), alongside multivariate indices Genotype-by-Trait (GT), Genotype-by-Yield × Trait (GYT), and Multi-Trait Genotype-Ideotype Distance Index (MGIDI) to facilitate multi-trait selection across marketable yield (MY), days to harvest (DTH), average bulb weight (ABW), total soluble solids (TSS), double bulb formation (DB), and thrips incidence (TI).

**Results:**

Pooled ANOVA and GGE analyses indicated significant genotype, environment and GEI effects for all traits. The first two GGE axes captured substantial variation of 56.7% (MY) to 86.6% (DB) of variation, supporting reliable biplot interpretation. Across complementary models, Bhima Shakti (G24), RO-1672 (G11), Bhima Kiran (G23) and RO-1773 (G19) consistently ranked among the top performers, exhibiting desirable stability profiles; MGIDI index further supported these selections. GGE delineated three mega environments for MY, DTH and TI, with vertex genotypes varying by trait, while AMMI biplots identified genotypes proximate to the origin as broadly stable. Environment ranking emphasized E6 (ICAR-DOGR, Pune) as most informative (discriminative and representative) for MY.

**Discussion/Conclusion:**

Integrating BLUP with AMMI, GGE and multi-trait selection indices enhances accuracy of genotype recommendations, enabling the identification of high-performing and stable red onion cultivars across diverse Indian agro-ecologies.

## 1 Introduction

Onion (*Allium cepa* L.) is one of the world’s most widely cultivated and consumed vegetables, valued for its characteristic pungency, culinary versatility, and documented therapeutic properties (Gupta et al., 2025a, 2025b; Pareek et al., 2017; Singh and Khar, 2022). Indian onions are particularly renowned for their pungency and occur in a wide range of bulb colors—red, purple, pink, yellow, and white—many of which are associated with specific culinary uses and consumer preferences (Gupta et al., 2025b; Gokce et al., 2010). Red onions, in particular, are preferred by Indian consumers for everyday cooking, salads, chutneys, and pickles because of their color, flavor, and perceived freshness, and they also represent an important export class. In several production regions, red-skinned types command premium prices due to their favorable organoleptic and storage attributes, making varietal choice a key determinant of farmer income and marketability (Hassan, 2015).

In India, onion is cultivated in three cropping seasons—*kharif* (June–November), *late kharif* (August–March), and *rabi* (October–May)—to ensure year-round availability. The *rabi* crop accounts for nearly 60% of national production, while *kharif* and *late kharif* crops each contribute roughly 20% (Hiremath and Mantur, 2018). The crop is grown across most parts of the country, except in some regions of the northeast and Kerala (Dhotre et al., 2025). Onion’s substantial genetic variability provides opportunities for breeding; however, bulb yield, a complex polygenic trait influenced by bulb weight, maturity, and plant architecture, remains vulnerable to biotic stresses (purple blotch, anthracnose, *Stemphylium* blight, thrips) and abiotic stresses (drought, salinity, temperature extremes), as well as postharvest losses (Barnoh, 2021; Chaudhry et al., 2023; Ratnarajah and Gnanachelvam, 2021; Muhie, 2022). Although molecular breeding and high-throughput phenotyping have accelerated trait discovery (Sharma et al., 2024), environmental variability still strongly affects yield, quality, and storability, leading to high genotype × environment interaction (GEI)–driven inconsistencies in performance across locations (Gupta et al., 2024b; Khokhar, 2017).

To resolve such inconsistencies, multi-environment trials (METs) are essential for robust genotype evaluation and cultivar recommendation, enabling the identification of genotypes that combine high productivity with broad or specific adaptability (DeLacy et al., 1996). However, pervasive GEI complicates selection, as genotypic responses often change with location and season (Romagosa et al., 2013). Graphical-analytic methods such as the Additive Main effects and Multiplicative Interaction (AMMI) model and the Genotype and Genotype × Environment (GGE) biplot are particularly useful because they jointly represent genotype main effects and GEI in a single ordination space, facilitating intuitive visualization of “which-won-where” patterns and stability relationships (Gauch, 2006; Yan and Tinker, 2006).

AMMI decomposes GEI into interpretable interaction principal components, while best linear unbiased predictions (BLUPs) obtained via restricted maximum likelihood (REML) provide shrinkage-adjusted estimates of genotypic merit that accommodate unbalanced designs and heterogeneous error variances (Ajay et al., 2018; Farshadfar, 2008; Jambhulkar et al., 2014; Olivoto et al., 2019; Zali et al., 2012). Treating genotypic effects as random, indices such as the harmonic mean of the relative performance of genotypic values (HMRPGV) allow simultaneous selection for productivity, stability, and adaptability (De Resende and De Resende, 2002; Dos Santos et al., 2019). More recent multivariate tools, including the GYT (genotype-by-yield x trait) biplot and the MGIDI (multi-trait genotype-ideotype distance index) index, further enhance selection efficiency by integrating yield stability with multiple trait profiles (Yan and Fregeau-Reid, 2018; Olivoto and Nardino, 2021).

Despite these advances, previous studies on red onion have often been limited by the evaluation of a small number of genotypes, restricted environmental coverage, or single-trait selection approaches, which constrain the identification of broadly adapted and stable genotypes. Moreover, the effects of GEI on multi-trait performance and yield stability are not always fully captured by conventional methods, limiting actionable recommendations for breeders and stakeholders.

In this context, the present study evaluated 24 red onion genotypes across eight diverse *rabi*-season environments in India. By integrating AMMI, GGE biplot, and BLUP analyses with contemporary multi-trait selection indices including HMRPGV, MGIDI, and GYT, the study aimed to (i) quantify GEI and partition its components, (ii) identify high-yielding genotypes with broad or specific adaptation, and (iii) provide robust, evidence-based recommendations for breeding and targeted cultivar deployment. This integrated analytical framework combines graphical interpretation, interaction partitioning, and robust prediction to deliver both intuitive visuals and statistically sound rankings for genotype selection, addressing key limitations of prior studies and supporting enhanced productivity and stability in red onion improvement.

## 2 Materials and methods

### 2.1 Experimental site

The study was conducted during two consecutive *rabi* seasons (2023–2024) at eight agro-climatically diverse test locations in India. Since the same locations were used in both years, these were considered as eight fixed environments (E1–E8) for analysis rather than as 16 separate location–year combinations. The test sites included the ICAR–Indian Agricultural Research Institute (ICAR-IARI), New Delhi; Regional Research Station of the National Horticultural Research and Development Foundation (RRS-NHRDF), Karnal; Chandra Shekhar Azad University of Agriculture and Technology (CSAUAT), Kanpur; Jawaharlal Nehru Krishi Vishwavidyalaya (JNKVV), Jabalpur; Junagadh Agricultural University (JAU), Junagadh; ICAR–Directorate of Onion and Garlic Research (ICAR-DOGR), Pune; ICAR–Indian Institute of Horticultural Research (ICAR-IIHR), Bengaluru; and Tamil Nadu Agricultural University (TNAU), Coimbatore (**Table 1**). The seasonal weather parameters recorded at these locations are presented in **Supplementary File 1**.

**Table 1:** Geographic details of the eight research environments (E1-E8) representing fixed locations evaluated across two consecutive *rabi* seasons (2023-24).

Sl. No.	Code	Location	Latitude/longitude
1.	E1	ICAR-IARI, New Delhi	28°38’23N, 77°09’27E
2.	E2	RRS, Karnal	29°74’86N/76°99’69E
3.	E3	CSAUAT, Kanpur	26°29’28.39 N/80°18’25.24E
4.	E4	JNKVV, Jabalpur	23°21’190.19N/79°95’884.35E
5.	E5	JAU, Junagadh	21°50’63 N/70°45’02E
6.	E6	ICAR-DOGR, Pune	27° 19’00.2N/82°25’00.1E
7.	E7	ICAR-IIHR, Bengaluru	13°13’48N/77°49’60E
8.	E8	TNAU, Coimbatore	11°01’2.2/76°93’5.4E

### 2.2 Plant materials and field trials

The experimental material comprised 24 red onion genotypes, including two checks (**Table 2**). Field trials were conducted during the *rabi* season of 2023–2024 across eight agro-ecologically distinct locations, following a randomized complete block design (RCBD) with three replications. Standard land preparation and crop management practices were adopted as per the guidelines of the ICAR–Directorate of Onion and Garlic Research (ICAR-DOGR), Pune. Transplanting was carried out using 45-day-old seedlings raised on nursery beds. Seedlings were established on raised beds measuring 1.0 m in width and 15 cm in height, with spacing of 15 × 10 cm (row × plant). Each replicate consisted of 70 plants, from which five plants were randomly sampled for trait evaluation, giving a total of 15 plants per genotype at each location. Harvesting was undertaken when more than 50% of the plants exhibited neck fall, indicating physiological maturity.

**Table 2:** List of 24 red onion genotypes included in this study.

Code	Genotype	Source
G1	RO-1619	ICAR-DOGR, Pune
G2	RO-1620	ICAR-DOGR, Pune
G3	RO-1621	ICAR-DOGR, Pune
G4	RO-1622	ICAR-DOGR, Pune
G5	RO-1625	ICAR-DOGR, Pune
G6	RO-1642	ICAR-DOGR, Pune
G7	RO-1654	ICAR-DOGR, Pune
G8	RO-1657	ICAR-DOGR, Pune
G9	RO-1664	ICAR-DOGR, Pune
G10	RO-1665	ICAR-DOGR, Pune
G11	RO-1672	ICAR-DOGR, Pune
G12	RO-1741	ICAR-DOGR, Pune
G13	RO-1747	ICAR-DOGR, Pune
G14	RO-1751	ICAR-DOGR, Pune
G15	RO-1757	ICAR-DOGR, Pune
G16	RO-1758	ICAR-DOGR, Pune
G17	RO-1769	ICAR-DOGR, Pune
G18	RO-1770	ICAR-DOGR, Pune
G19	RO-1773	ICAR-DOGR, Pune
G20	RO-1783	ICAR-DOGR, Pune
G21	RO-1784	ICAR-DOGR, Pune
G22	RO-1824	ICAR-DOGR, Pune
G23	Bhima Kiran	ICAR-DOGR, Pune
G24	Bhima Shakti	ICAR-DOGR, Pune

### 2.3 Phenotypic data collection

Observations were recorded for six key traits: (i) marketable yield (q/ha), (ii) days to harvest, (iii) average bulb weight (g), (iv) total soluble solids (TSS, %), (v) double bulb formation (%), and (vi) thrips incidence (%). Marketable yield was estimated from the total weight of harvestable bulbs from all plants within each plot and expressed on a per-hectare basis. Average bulb weight was calculated as the mean weight of marketable bulbs from the sampled plants, while TSS content was determined using a hand-held refractometer. Thrips incidence was assessed visually using a 1–5 severity rating scale adapted from Smith et al. (1994), where 1 = 1%–20% foliage damage, 2 = 21%–40%, 3 = 41%–60%, 4 = 61%–80%, and 5 = 81%–100%. The percentage of double bulbs was computed as the proportion of sampled bulbs exhibiting splitting or twin-bulb formation.

### 2.4 Statistical analysis

All statistical analyses and graphical outputs were performed in R (version 2025.05.1) using the metan package. Data handling and plotting followed standard procedures for multi-environment trial (MET) analysis. Unless stated otherwise, tests were conducted at the 5% significance level. Data from each environment were inspected for entry errors and missing values, and summary statistics (means, standard deviations, coefficients of variation) were computed. Homogeneity of residual variances across environments was tested by Bartlett’s test (α = 0.05). Individual analyses of variance (ANOVA) were then performed separately for each environment to assess within-site genotypic variation. When Bartlett’s test indicated homogeneous variances, a combined ANOVA across environments was fitted to partition total variation into environment (E), genotype (G), and genotype × environment interaction (G×E) components. The combined ANOVA was based on the linear model (Equation 1):


(1) yijk=μ+gi+ej+(ge)ij+rk(j)+ϵijk

where, *yijk* is the observed plot mean for genotype *i* in *j* environment and replication *k*; μ is the overall mean; *gi* is the genotype effect; *ej* is the environment effect; *(ge)ij* is the genotype × environment interaction; *rk(j)* is the replication effect nested within environment; and *εijk*; is the residual error. For the combined ANOVA reported in the results, genotypes and environments were treated as fixed effects to summarize observed performance and interaction patterns.

Best linear unbiased predictions (BLUPs) of genotypic effects were obtained from linear mixed models in which genotypes (and, where appropriate G×E) were modeled as random effects, and variance components were estimated by restricted maximum likelihood (REML). The mixed model used for BLUP estimation corresponded to the combined model above but with *g* (and/or (*ge*)*ij* depending on the specific analysis) treated as random. BLUPs were used to generate shrinkage-adjusted genotype means, rank genotypes, and compute prediction standard errors.

The additive main effects and multiplicative interaction (AMMI) and genotype × environment interaction (GGE) biplot models were used to visualize the winning genotypes and mega-environments (MEs) for yield-related traits (Yan et al., 2000). To support multi-trait selection, we computed the harmonic mean of the relative performance of genotypic values (HMRPGV) following De Resende and De Resende (2002) and the multi-trait genotype-ideotype distance index (MGIDI) as described by Olivoto and Nardino (2021), as follows (Equation 2):


(2) $$MGIDI_{i}=\sqrt{{\sum_{j=1}^{f}\left(\gamma_{ij}-\gamma_{j}\right)}^{2}}$$

where, $\gamma_{ij}\;$ is the score of the *i^th^* genotype on the *j^th^* factor (obtained from factor analysis or PCA), $\gamma_{j}\;$ is the ideotype score for factor *j*, *f* is the number of retained factors, *i*= 1, 2, …, *g* indexes genotypes and *j* = 1, 2, ……, *f* indexes the factors. Prior to MGIDI computation, traits were oriented so that the desired direction (increase or decrease) was consistent, and factor scores were standardized when required by the method.

We also employed genotype-by-yield × trait (GYT) biplots to visualise multi-trait performance. For AMMI and GGE, the number of interaction axes retained for interpretation was chosen based on the proportion of interaction variance explained and graphical clarity. For factor-based index (MGIDI), the number of factors retained followed Kaiser’s criterion (eigenvalues > 1) and examination of the scree plot. For all models, estimated variance components, BLUPs, mean squares, F-values and the percentage variance explained by model terms or axes are reported in the results together with the associated significance levels.

## 3 Results

Pooled ANOVA indicated significant differences (*P* < 0.05) among genotypes across environments (**Supplementary File 2**), confirming substantial genetic variability. Based on pooled means, Bhima Shakti achieved the highest yield (330.34 q/ha), followed by Bhima Kiran (302.23 q/ha), while RO-1824 recorded the lowest (211.33 q/ha). Similarly, RO-1620 and RO-1622 were earliest to harvest, whereas Bhima Kiran was late maturing. Detailed descriptive performance across environments is provided in **Supplementary Files 2, 3**.

### 3.1 BLUP-based summary of yield-related traits across environments

To obtain more robust rankings that account for environmental variation, BLUP-based stability indices were computed (**Table 3**). Across traits, Bhima Shakti (G24), Bhima Kiran (G23), RO-1769 (G17), RO-1773 (G19), and RO-1672 (G11) emerged as consistently high- and stable-yielding genotypes. For days to harvest, RO-1620 (G2), RO-1622 (G4), and RO-1619 (G1) matured earliest, while for average bulb weight, Bhima Shakti (G24), RO-1672 (G11), Bhima Kiran (G23), RO-1773 (G19), and RO-1620 (G2) were superior. Regarding TSS, RO-1784 (G21), Bhima Kiran (G23), and Bhima Shakti (G24), along with RO-1654 (G7), recorded the highest values. For double-bulb formation, most genotypes showed above-mean values, although RO-1769 (G17) and RO-1770 (G18) consistently displayed the lowest. For thrips incidence, RO-1642 (G6), RO-1751 (G14), and RO-1654 (G7) were identified as the most resistant.

**Table 3:** HMRPGV-based BLUP scores for onion yield-related traits.

GEN	MY (q/ha)	Rank	DTH (days)	Rank	ABW (g)	Rank	TSS (°Brix)	Rank	DB (%)	Rank	TI (scale 1-5)	Rank
G24	1.28	1	1.01	19	1.17	1	1.02	3	0.00	–	1.04	20
G23	1.18	2	1.02	24	1.08	3	1.03	2	0.00	–	1.04	21
G17	1.08	3	1.01	20	1.00	10	0.99	15	0.00	–	1.01	16
G19	1.08	4	1.02	23	1.06	4	0.99	14	0.00	–	1.01	14
G11	1.03	5	1.00	17	1.11	2	1.01	7	0.00	–	1.04	22
G1	1.03	6	0.98	3	1.02	9	1.01	9	0.00	–	1.01	17
G7	1.02	7	1.00	13	0.99	11	1.02	4	0.00	–	0.91	3
G18	1.01	8	1.01	21	1.02	8	1.00	12	0.00	–	1.01	15
G8	1.00	9	1.01	18	1.03	7	1.01	6	0.00	–	1.00	10
G15	0.97	10	0.99	8	0.89	23	1.00	13	0.00	–	0.93	5
G16	0.95	11	0.99	7	0.98	12	1.00	11	0.00	–	1.00	11
G14	0.94	12	1.00	10	0.91	22	0.98	18	0.00	–	0.89	2
G5	0.93	13	1.00	9	0.95	14	0.97	22	0.01	–	0.93	4
G2	0.92	14	0.97	1	1.05	5	0.98	19	0.00	–	1.06	24
G6	0.92	15	0.99	6	0.96	13	1.01	5	0.00	–	0.89	1
G21	0.90	16	1.02	22	1.03	6	1.04	1	0.00	–	0.96	6
G12	0.90	17	1.00	12	0.92	18	0.96	24	0.00	–	1.00	9
G13	0.86	18	1.00	16	0.93	17	0.96	23	0.00	–	0.98	8
G20	0.85	19	1.00	15	0.92	19	0.98	17	0.00	–	1.00	12
G4	0.84	20	0.98	2	0.91	21	1.01	8	0.00	–	1.03	18
G10	0.81	21	1.00	11	0.95	16	0.98	20	0.00	–	1.06	23
G9	0.80	22	0.99	4	0.95	15	1.01	10	0.00	–	0.98	7
G22	0.77	23	1.00	14	0.92	20	0.98	16	0.00	–	1.01	13
G3	0.72	24	0.99	5	0.87	24	0.98	21	0.00	–	1.03	19

GEN, Genotype; MY, marketable yield; DTH, days to harvest; ABW, average bulb weight; TSS, total soluble solids; DB, double bulb formation; TI, thrips incidence

Overall, BLUP results largely supported pooled mean performance while refining genotype rankings, thereby providing a more reliable basis for selecting genotypes with wide adaptation (Bhima Shakti, Bhima Kiran, RO-1672), as well as those with specific advantages such as early maturity (RO-1620), low double-bulb incidence (RO-1769, RO-1770), and thrips resistance (RO-1751, RO-1642). Trait-wise, the top-performing environments were as follows: E6 > E7 > E5 for marketable yield; E4 > E6 > E2 for days to harvest; E7 > E6 > E5 for average bulb weight; E5 > E2 > E3 for TSS; E1 = E8 > E5 for double bulbs; and E7 > E2 = E3 = E4 = E8 for thrips incidence (**Supplementary File 3**).

### 3.2 Genotype evaluation using GGE biplot

**Figure 1:**
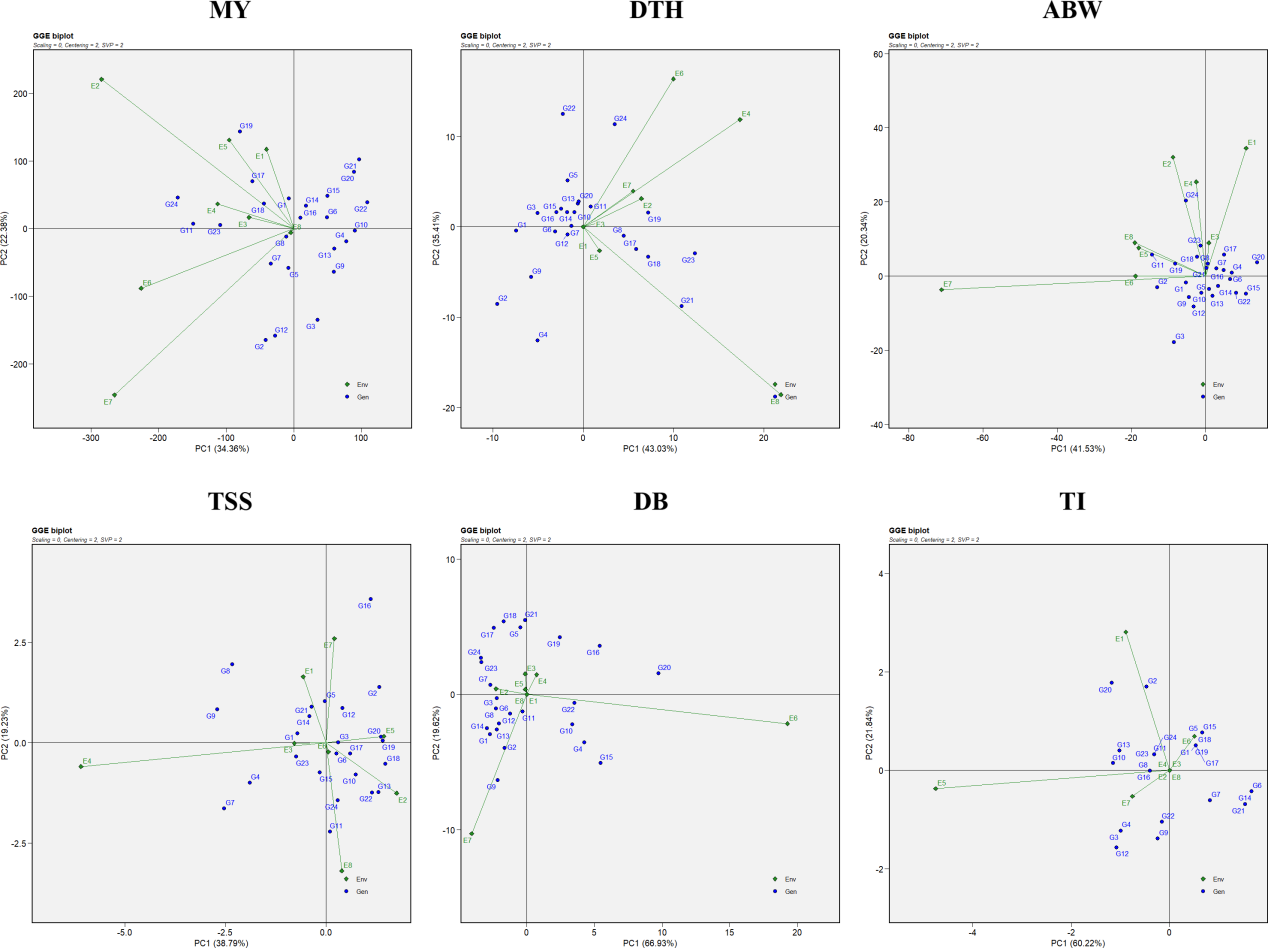
GGE biplot representation of onion genotypes for the evaluated traits: MY, marketable yield; DTH, days to harvest; ABW, average bulb weight; TSS, total soluble solids; DB, double bulb formation; and TI, thrips incidence.

The combined ANOVA based on the GGE biplot approach revealed statistically significant differences (*P* < 0.05) among genotypes, environments, and their interactions (G × E) for all six evaluated traits: marketable yield, days to harvest, average bulb weight, TSS, double bulbs, and thrips incidence (**Table 4**). The biplot analysis effectively explained a considerable proportion of the total variation through the first two principal components (PC1 and PC2), with cumulative variation ranging from 55% to 85% depending on the trait. Specifically, the proportion of variation explained by PC1 and PC2 was 56.74% for marketable yield, 78.44% for days to harvest, 61.87% for average bulb weight, 58.02% for TSS, 86.55% for double bulbs, and 82.06% for thrips incidence. Thus, the first two principal components successfully captured a substantial share of the total variability across the eight environments (**Figure 1**).

**Table 4:** Combined analysis of variance using GGE biplot approach.

Source of variation	Df	Marketable yield		Days to harvest		Average bulb weight		TSS		Double bulbs		Thrips incidence	
		MSS	SS (%)	MSS	SS (%)	MSS	SS (%)	MSS	SS (%)	MSS	SS (%)	MSS	SS (%)
ENV	7	823101.99*	73.96	2742.09*	66.05	33236.36*	83.23	180.82*	77.45	325.75*	56.35	81.87*	66.20
REP (ENV)	16	80.87	0.02	4.86	0.27	2.73	0.02	0.09	0.09	0.00	0.00	0.57	1.05
GEN	23	21767.49*	6.43	69.46*	5.50	488.54*	4.02	1.53*	2.16	6.08*	3.45	0.77*	2.03
GEN: ENV	161	9367.33*	19.36	31.46*	17.43	213.81*	12.31	1.87*	18.42	10.09*	40.13	0.64*	11.90
Error	368	50.80	0.24	8.50	10.76	3.21	0.42	0.08	1.88	0.01	0.07	0.44	18.82

*Indicate significance at *P* < 0.05; Df, degree of freedom; SS, sum of squares; MS, mean square; ENV, environment; REP, replication; GEN, genotype; GEN: ENV, genotype × environment interaction

#### 3.2.1 Stability assesment using average environment coordination view

**Figure 2:**
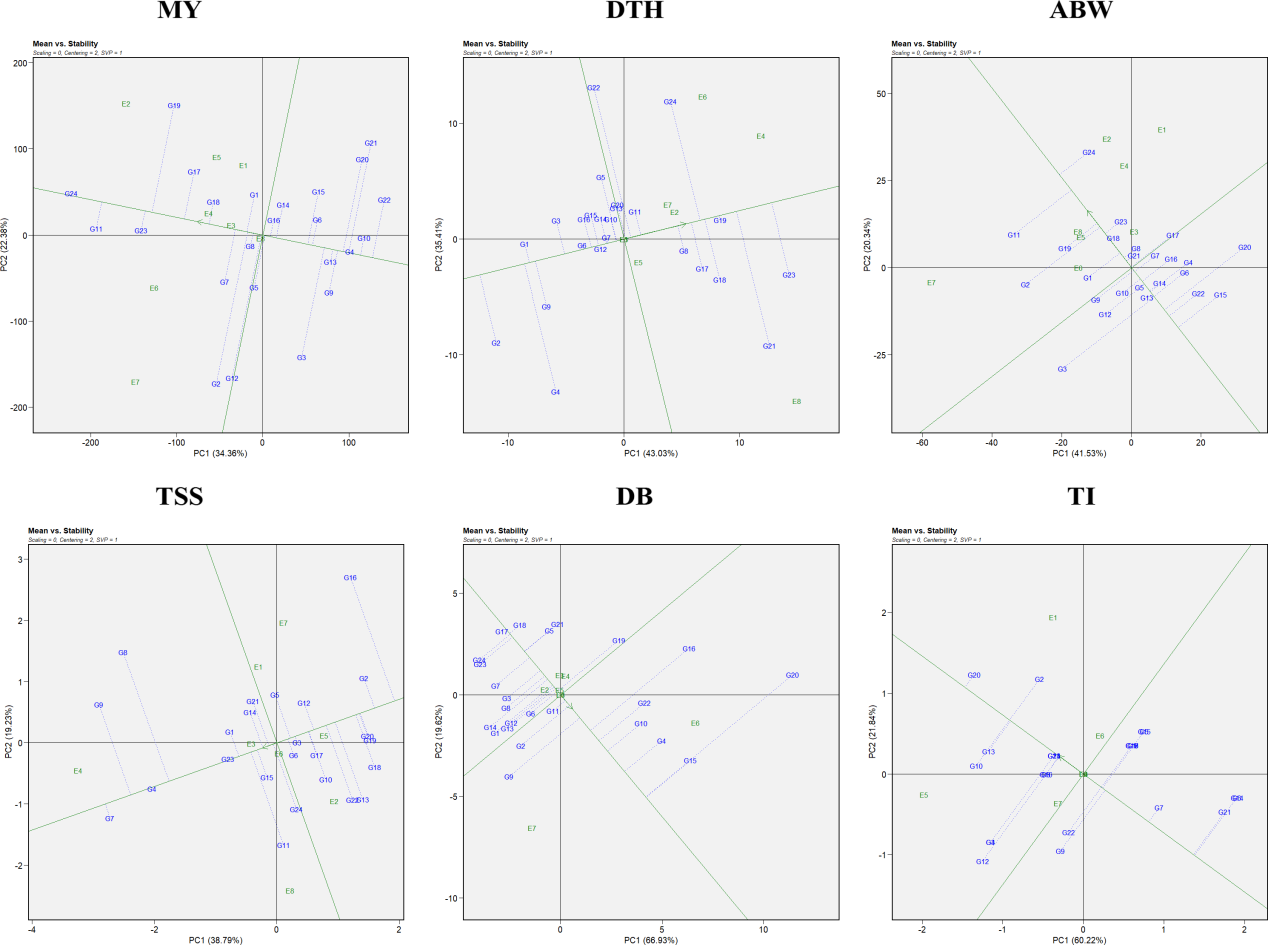
Mean vs stability representation of the GGE biplot for evaluated traits: MY: marketable yield; DTH, days to harvest; ABW, average bulb weight; TSS, total soluble solids; DB, double bulb formation; and TI, thrips incidence.

Genotypic performance and stability were evaluated using the average environment coordination (AEC) view of the GGE biplot (**Figure 2**). The horizontal axis (x-axis) represents the average performance of genotypes across environments, derived from the mean PC1 and PC2 scores. The arrow along this axis points toward genotypes with higher average performance. The vertical axis (y-axis), perpendicular to the average environment axis, reflects genotype stability, with genotypes located closer to this axis considered more stable and those farther away regarded as less stable (Yan and Tinker, 2006). For marketable yield, the most stable genotypes included G4 (RO-1622), G24 (Bhima Shakti), G13 (RO-1747), and G8 (RO-1657). Notably, genotypes G24 (Bhima Shakti), G11 (RO-1672), G23 (Bhima Kiran), and G18 (RO-1770) combined both high yield potential and stability.

In the case of days to harvest, the most stable genotypes were G6 (RO-1642), G12 (RO-1741), G19 (RO-1773), and G7 (RO-1654), while G1 (RO-1619), G6 (RO-1642), G12 (RO-1741), and G7 (RO-1654) demonstrated both earliness and stability across environments. For average bulb weight, stability was greatest in G18 (RO-1770), G5 (RO-1625), and G13 (RO-1747). Notably, genotypes G24 (Bhima Shakti), G19 (RO-1773), G23 (Bhima Kiran), and G18 (RO-1770) emerged as both productive and stable. For TSS, genotypes G4 (RO-1622), G23 (Bhima Kiran), and G3 (RO-1621) were identified as the most stable, whereas G7 (RO-1654), G4 (RO-1622), and G23 (Bhima Kiran) excelled in both performance and stability. For double-bulb formation, genotypes G17 (RO-1769), G18 (RO-1770), G24 (Bhima Shakti), and G23 (Bhima Kiran) had the lowest values, with G17 (RO-1769), G18 (RO-1770), and G11 (RO-1672) being both stable and superior in performance. For thrips incidence, G23 (Bhima Kiran) and G24 (Bhima Shakti) demonstrated high stability. Notably, G7 (RO-1654), G23 (Bhima Kiran), and G24 (Bhima Shakti) were identified as ideal genotypes with both low thrips incidence and consistent performance.

#### 3.2.2 Mega-environment analysis

**Figure 3:**
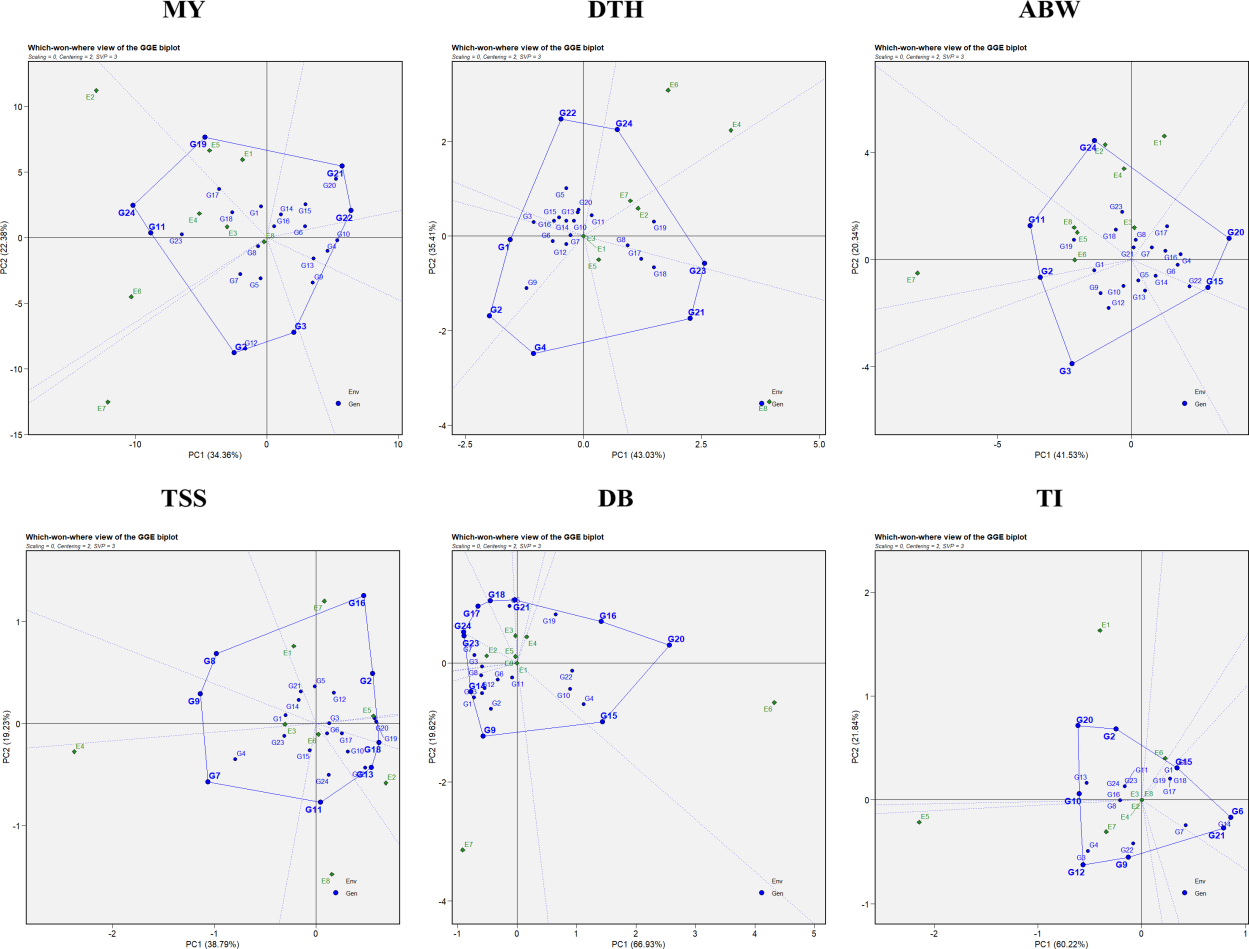
Which-won-where polygon view of the GGE biplot for evaluated traits: MY: marketable yield; DTH: days to harvest; ABW: average bulb weight; TSS: total soluble solids; DB: double bulb formation; and TI: thrips incidence.

The “which-won-where” pattern of the GGE biplot facilitates visualization of genotype performance across multiple environments by dividing the biplot into sectors, each representing a different mega-environment (**Figure 3**). The polygon was constructed by connecting genotypes that were farthest from the biplot origin, with vertex genotypes representing either the highest or lowest performance in specific environments. Equality lines perpendicular to the polygon sides delineated the sectors and enabled the identification of winning genotypes for each group of environments. Based on this analysis, three mega-environments were identified for marketable yield, days to harvest, and thrips incidence; two mega-environments for average bulb weight; five mega-environments for TSS; and four mega-environments for double-bulb formation.

For marketable yield, environments E7 and E8 grouped into the first mega-environment with G2 and G3 as winners; E2, E3, E4, and E6 formed the second mega-environment with G11 and G24 as winners, while E1 and E5 constituted the third mega-environment with G19 as the top performer. For days to harvest, E1, E3, E5, and E8 formed the first mega-environment, where G21 was the best performer; E2 represented the second mega-environment with G23 as winner; and the remaining environments clustered into a third mega-environment with G24 as winner. For average bulb weight, E5, E6, E7, and E8 grouped into one mega-environment dominated by G11, whereas the remaining environments formed a second mega-environment where G24 excelled.

For TSS, five distinct mega-environments were observed: E1, E5, and E7 grouped together with G2 and G16 as winners; E3 represented a single-environment cluster with G9 as winner; E4 formed another with G7; E6 and E8 clustered together with G11 as the best, while E2 grouped separately with G13 and G18 as winners. For double-bulb formation, four mega-environments were defined: E1, E7, and E8 grouped together with G9 and G14 as winners; E4 and E6 formed another with G15, G16, and G20; E3 and E5 clustered together with G18 and G21; and E2 represented the fourth with G23 and G24. For thrips incidence, E2, E3, E4, E5, E7, and E8 grouped into a broad mega-environment where G9 and G12 performed best; E1 formed a second mega-environment with G2, G10, and G20 as winners, while E6 represented a third cluster with G15 as winner.

#### 3.2.3 Evaluating ideal environments: discriminativeness vs representativeness

Assessment of an ideal environment is fundamental for identifying superior genotypes adapted to specific multi-environments (MEs). Discriminativeness refers to an environment’s ability to distinguish among genotypes and is reflected by the length of its vector in the GGE biplot. Representativeness is determined by the angle between the environment’s vector and the AEC axis, with an acute angle signifying high representativeness. An ideal environment possesses both a long vector (high discriminativeness) and a small angle with the AEC axis (high representativeness).

**Figure 4:**
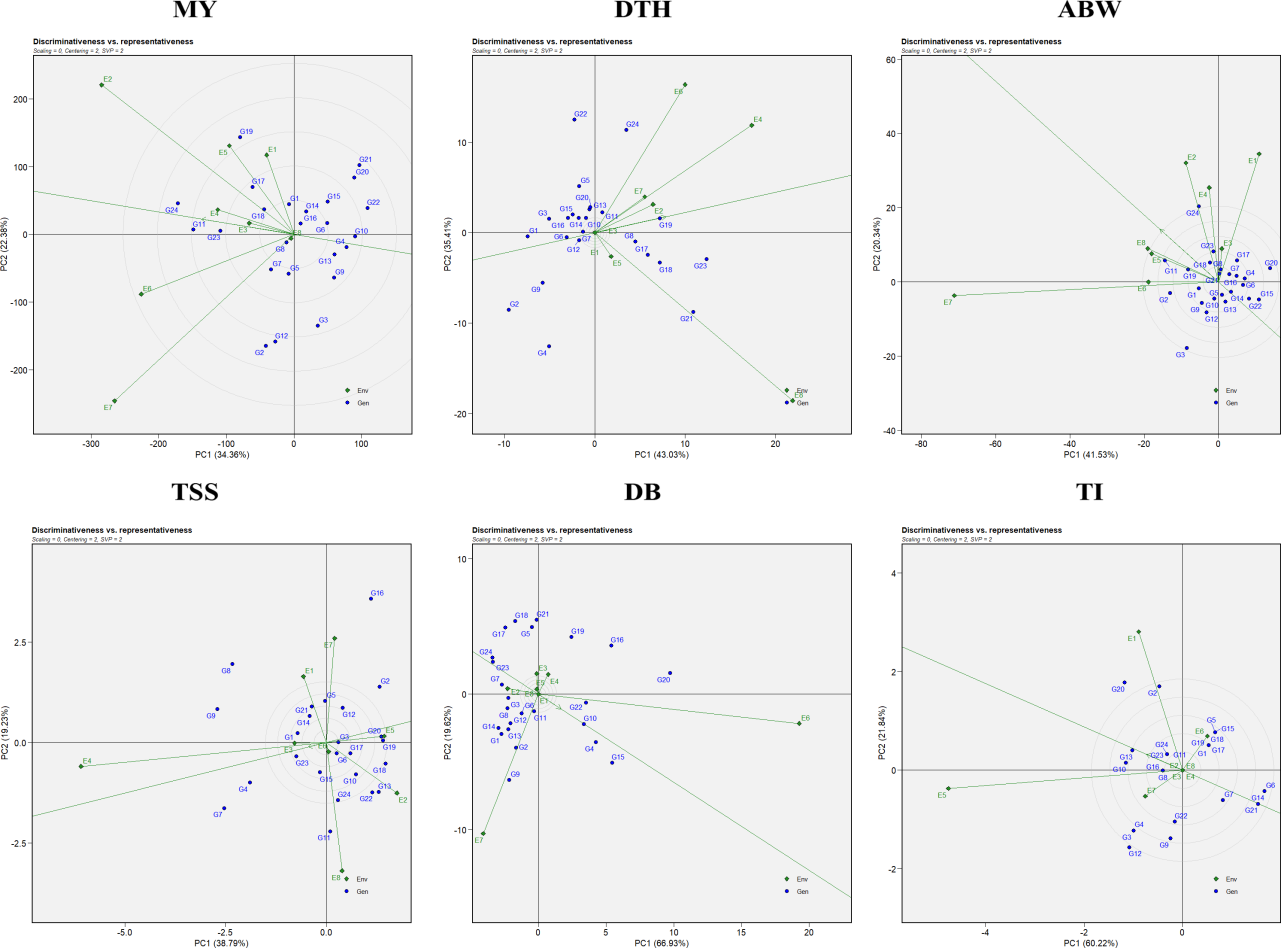
Discriminativeness vs. representativeness view of the GGE biplot for evaluated traits: MY, marketable yield; DTH, days to harvest; ABW, average bulb weight; TSS, total soluble solids; DB, double bulb formation; and TI, thrips incidence.

In this study, E6 emerged as the most effective environment for evaluating marketable yield and double-bulb formation; E4 for days to harvest and TSS; E5 for thrips incidence; and E8 for average bulb weight (**Figure 4**). These environments provided optimal conditions for selecting stable, well-adapted genotypes. In contrast, environments such as E7 (for marketable yield, average bulb weight, and double-bulb formation), E8 (for days to harvest and TSS), and E1 (for thrips incidence) were highly discriminative but less representative. Overall, E6 (ICAR–DOGR, Pune) and E4 (JNKVV, Jabalpur) were identified as the most suitable test environments for onion trait evaluation, offering both strong discrimination and reliable representativeness of overall performance trends.

#### 3.2.4 Ranking genotypes and environments using GGE biplot

**Figure 5:**
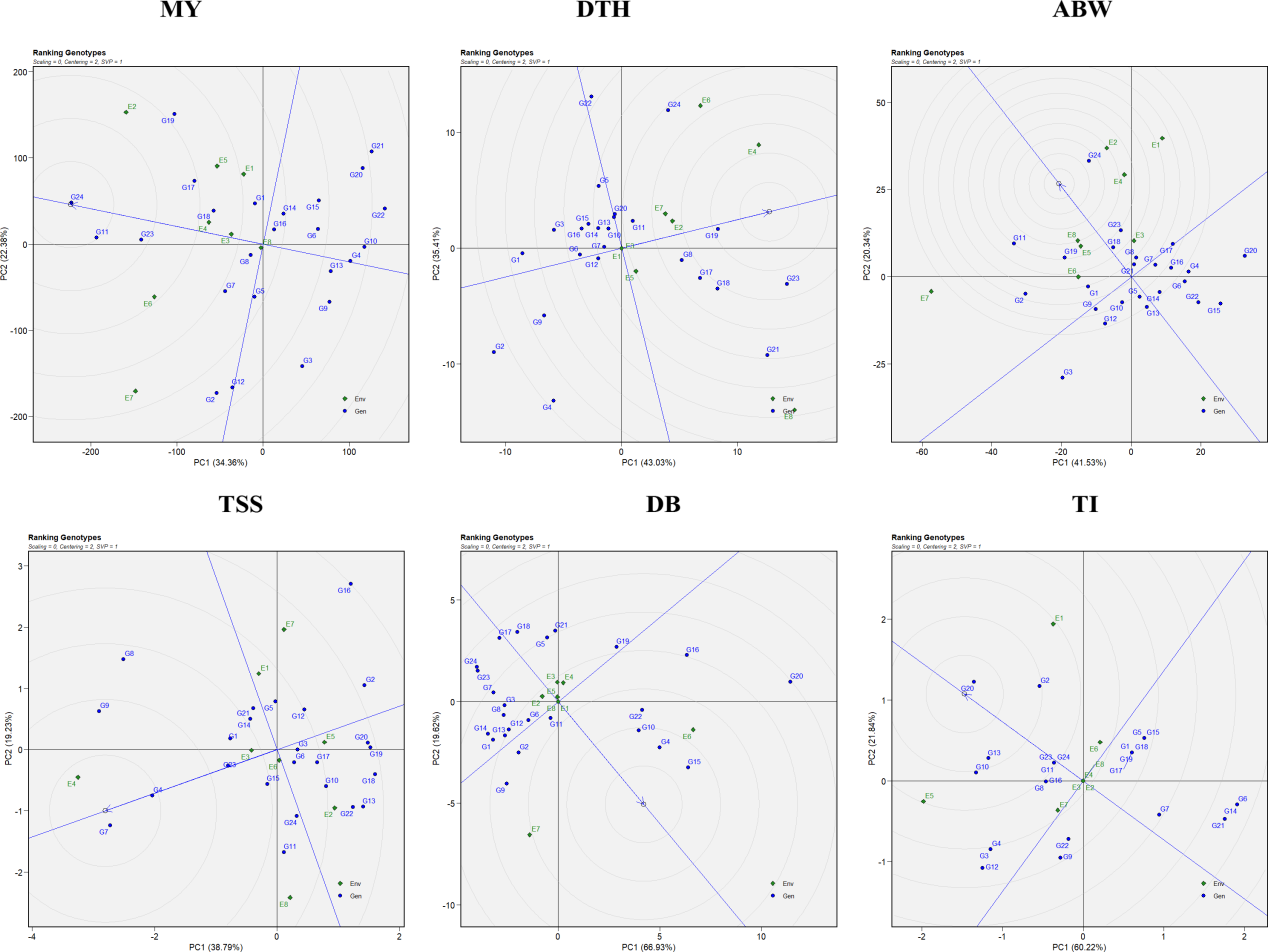
Ranking genotypes view of the GGE biplot for the evaluated traits: MY: marketable yield; DTH, days to harvest; ABW, average bulb weight; TSS, total soluble solids; DB, double bulb formation; and TI, thrips incidence.

The GGE biplot facilitates the identification of both superior genotypes and favorable test environments across multiple traits. An ideal genotype is defined as one that combines high mean performance with consistent stability across diverse environments. In the biplot (**Figure 5**), the ideal genotype is represented at the center of the innermost concentric circle, and genotypes positioned closer to this reference point are regarded as more suitable candidates for selection and breeding. For marketable yield, G24 (Bhima Shakti) was ranked as the ideal performer, followed by G11 (RO-1672), G23 (Bhima Kiran), G17 (RO-1769), and G19 (RO-1773). For days to harvest, G2 (RO-1620) was the earliest-maturing genotype, followed by G4 (RO-1622), while G19 (RO-1773) and G23 (Bhima Kiran) were late maturing. In terms of average bulb weight, G24 (Bhima Shakti) was closest to the ideal genotype, with G11 (RO-1672), G19 (RO-1773), and G23 (Bhima Kiran) showing competitive performance. For TSS, G7 (RO-1654) was the top-ranking genotype, followed by G4 (RO-1622), G9 (RO-1664), and G23 (Bhima Kiran). With respect to double-bulb percentage, G17 (RO-1769) and G18 (RO-1770) recorded the lowest (desirable) values, whereas G15 (RO-1757) and G4 (RO-1622) had higher (undesirable) values. For thrips incidence, genotypes G6 (RO-1642), G14 (RO-1751), and G21 (RO-1784) exhibited the highest resistance, while G20 (RO-1783) showed the highest incidence among all evaluated genotypes.

**Figure 6:**
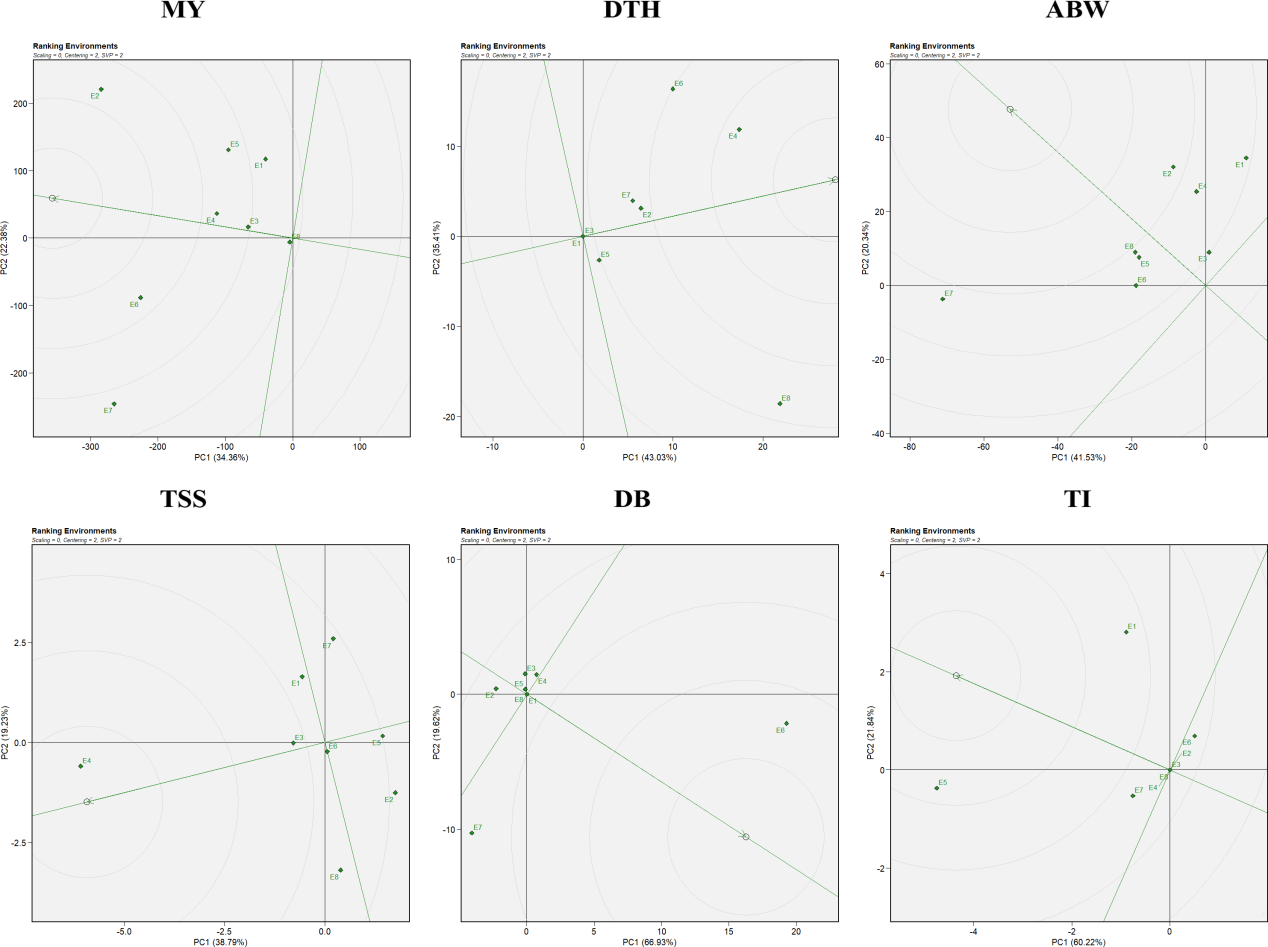
Ranking environments view of the GGE biplot for the evaluated traits: MY: marketable yield; DTH, days to harvest; ABW, average bulb weight; TSS, total soluble solids; DB, double bulb formation; and TI, thrips incidence.

In the environment-focused biplot, the arrow on the x-axis at the center of the concentric circles denotes the ideal environment (**Figure 6**). Test locations situated closer to this point are considered more desirable for evaluating genotypes. The relative distance of each environment from the ideal reference is reflected by its position along the concentric circles, providing a visual measure of its representativeness and discriminative ability. Regarding environments, E6, followed by E2 and E4, were closest to the ideal for marketable yield. For days to harvest, although E4 and E6 recorded higher values (indicating delayed maturity), E1, E3, and E5—being farthest from the delayed environments—were considered better for early maturity. For average bulb weight, E2, E8, and E4 were near the ideal environment, whereas for TSS, E4, E3, and E6 showed better suitability. For double-bulb formation, E2, E3, and E4 were farthest from the ideal environment and thus preferred for selecting genotypes with minimal expression of this trait. Similarly, for thrips incidence, E6, E2, E4, E3, and E8—being farthest from the ideal environment—were suitable for identifying resistant genotypes (**Figure 6**). Overall, E6 (ICAR–DOGR, Pune), E2 (RRS, Karnal), and E4 (JNKVV, Jabalpur) were identified as the most favorable environments, consistently supporting superior genotype performance across multiple traits.

### 3.3 Genotype-by-yield × trait biplot

**Figure 7:**
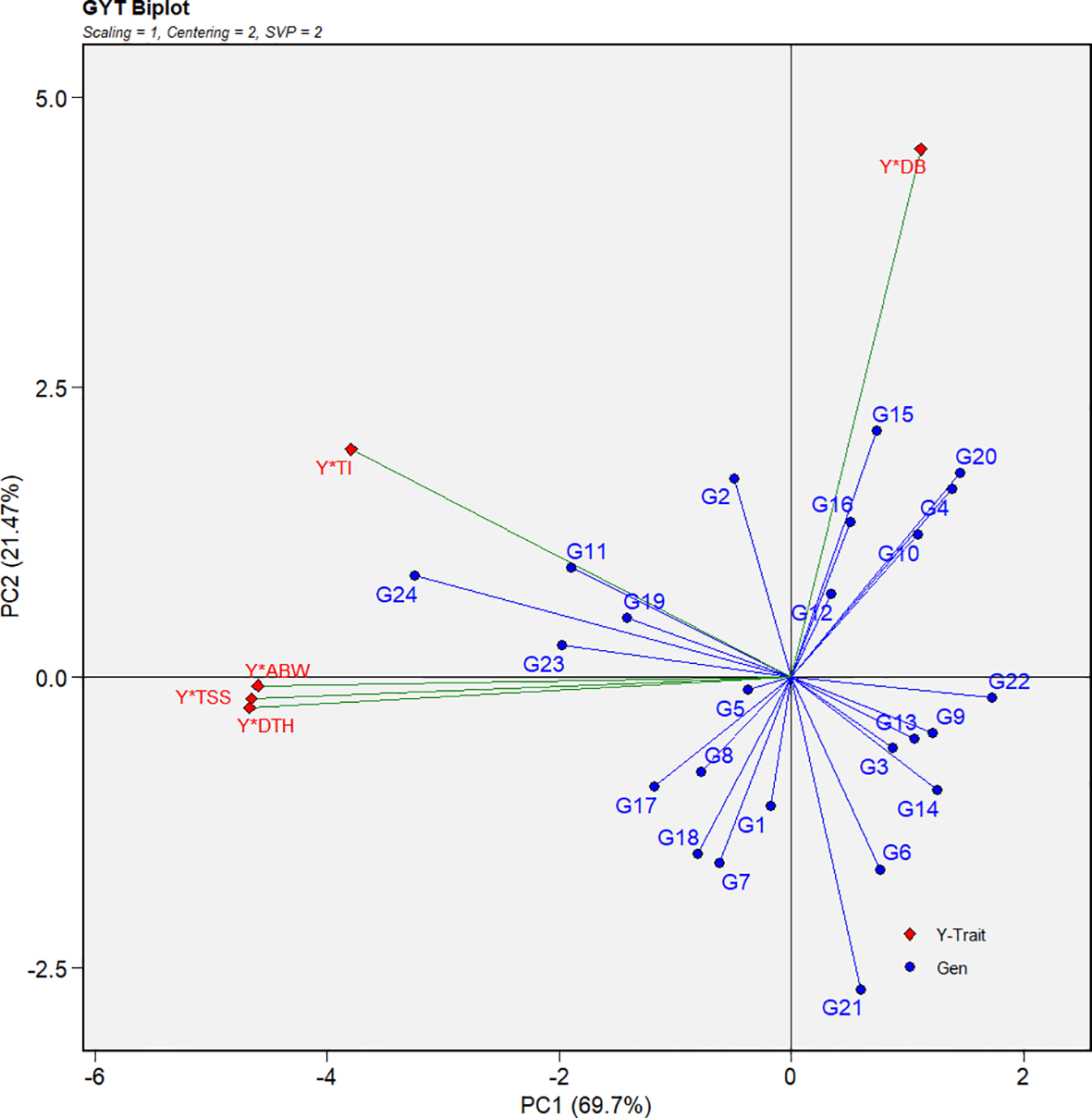
Genotype-by-yield × trait (GYT) biplot illustrating yield-trait associations and multi-trait performance for 24 red onion genotypes evaluated across eight *rabi* environments. Traits: DTH (days to harvest), ABW (average bulb weight), TSS (total soluble solids), DB (double bulb formation), and TI (thrips incidence).

The genotype-by-yield × trait (GYT) biplot offers a more integrated assessment than the genotype × trait (GT) biplot by combining yield with each trait, thereby enabling a comprehensive evaluation of genotypic performance (Yan and Fregeau-Reid, 2018). The biplot revealed predominantly positive correlations among the yield–trait combinations, as indicated by the acute angles formed between vectors (**Figure 7**). Notably, yield × average bulb weight, yield × total soluble solids, yield × days to harvest, and yield × thrips incidence displayed strong positive associations, indicating that genotypes performing well in these combinations are likely to be high yielding with favorable trait profiles. The only exception was yield × double-bulb formation, which did not show a positive relationship, suggesting its limited contribution to overall yield performance and its potential role as a negative selection trait.

### 3.4 Multi-trait genotype-ideotype distance index and factor analysis

The multi-trait genotype-ideotype distance index (MGIDI) is a robust multivariate selection tool that ranks genotypes based on their proximity to an ideotype—an ideal genotype that combines desirable performance across all target traits. By integrating multiple traits using principal component analysis, MGIDI facilitates the identification of genotypes with balanced and superior performance (Olivoto and Nardino, 2021).

**Figure 8:**
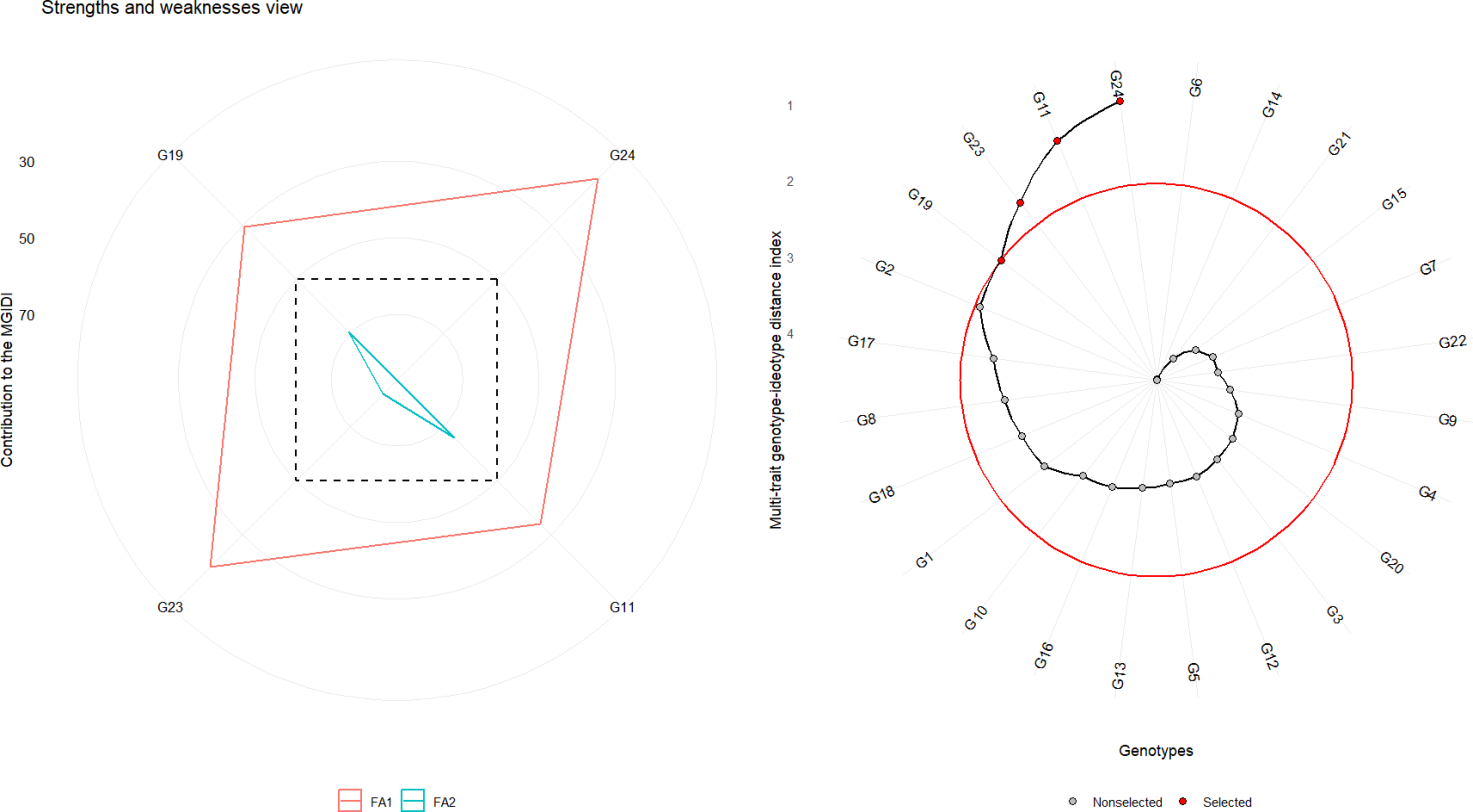
Multi-trait selection of 24 red onion genotypes across eight *rabi* environments using the MGIDI. Left panel: Strengths and weaknesses view showing the contribution of each trait to MGIDI for the first two factors (FA1 and FA2). Right panel: Genotype-ideotype distance plot, with genotypes closer to the red circle representing selections approaching the ideotype. Selected genotypes are shown in red dots; non-selected genotypes are shown in black dots.

MGIDI analysis identified G24 (Bhima Shakti), G11 (RO-1672), G23 (Bhima Kiran), and G19 (RO-1773) as the most promising genotypes (**Figure 8**). These genotypes, highlighted with red circles, exhibited the shortest distances from the ideotype, indicating high adaptability and superior performance across diverse environments. These selections suggest strong potential for improving overall onion productivity and trait integration.

The selection differential based on MGIDI revealed considerable variability in expected genetic gain across the studied traits (**Table 5**). High communalities were observed for average bulb weight and thrips incidence, followed by marketable yield and double-bulb formation, whereas the lowest communalities were recorded for days to harvest and TSS. High uniqueness was observed for TSS, followed by days to harvest, double-bulb formation, and marketable yield, whereas lower uniqueness was recorded for average bulb weight and thrips incidence. The selection differential was highest for marketable yield, followed by average bulb weight and days to harvest, and lowest for TSS and thrips incidence. The greatest genetic gains were observed for marketable yield (14.0%) and average bulb weight (8.34%), demonstrating the effectiveness of MGIDI-based selection in enhancing key productivity traits. Moderate improvements were noted in days to harvest (0.96%), thrips incidence (0.72%), and TSS (0.58%). Conversely, negative genetic gains were observed for double-bulb formation (–2.83%), which is advantageous since reductions in this trait are desirable for improving bulb quality. Overall, sense of gain was positive for all traits (**Table 5**).

**Table 5:** Factor analysis and selection differential for bulb yield and related traits in red onion genotypes.

Traits	Factor	FA1	FA2	Communality	Uniqueness	Xo	Xs	SD	SD (%)	h^2^	SG (%)	Sense	Goal
MY	FA1	-0.86	-0.16	0.77	0.23	257	299	41.3	16.1	0.871	14.0	increase	100
DTH	FA1	-0.67	0.18	0.48	0.52	124	125	1.52	1.23	0.782	0.958	increase	100
ABW	FA1	-0.88	-0.32	0.87	0.13	63.7	69.8	6.13	9.63	0.866	8.34	increase	100
TSS	FA1	-0.62	0.28	0.46	0.54	11.8	12	0.115	0.971	0.602	0.584	increase	100
DB	FA1	0.67	-0.39	0.59	0.41	1.43	1.35	-0.0793	-5.55	0.509	-2.83	increase	0
TI	FA2	0.01	-0.93	0.87	0.13	1.62	1.65	0.034	2.1	0.342	0.718	increase	100

MY, marketable yield; DTH, days to harvest; ABW, average bulb weight; TSS, total soluble solids; DB, double bulbs; TI, thrips incidence; Xo, mean of genotypes; Xs, mean of selected genotypes, SD, selection differential; SG, selection gain; FA, factor analysis; h2, heritability

Furthermore, genotypes G24 (Bhima Shakti), G23 (Bhima Kiran), G19 (RO-1773), and G11 (RO-1672) were closely associated with Factor 1 (FA1), which encapsulated favorable contributions from most traits except thrips incidence. Notably, none of the selected genotypes were linked to Factor 2 (FA2), which primarily represented high thrips incidence—an undesirable trait. This indicates that the top-performing genotypes not only excelled in productivity-related attributes but also displayed natural resistance to thrips.

### 3.5 AMMI biplot analysis

**Figure 9:**
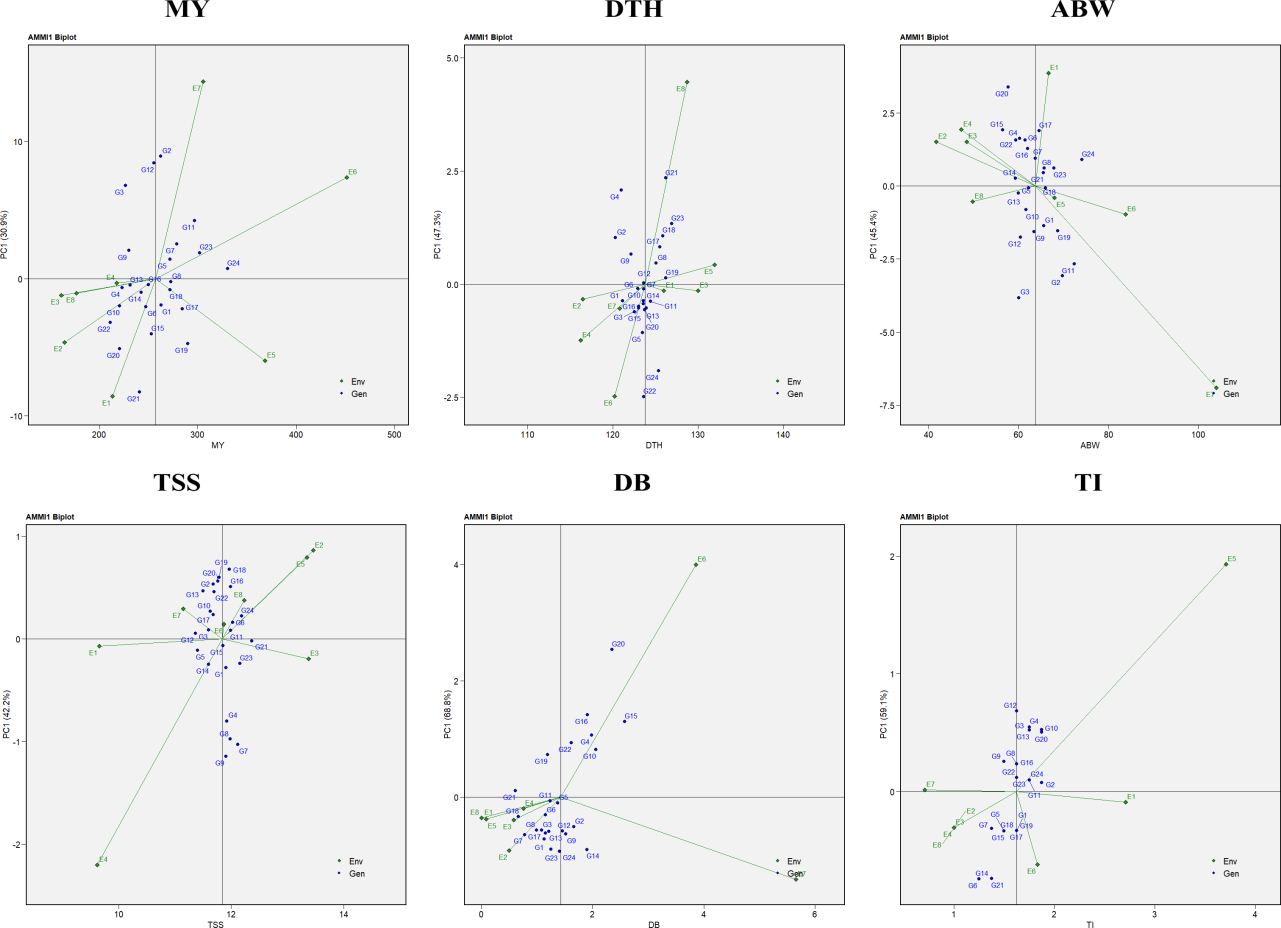
AMMI-1 biplot representation of onion genotypes across eight diverse environments for the evaluated traits: MY, marketable yield; DTH, days to harvest; ABW, average bulb weight; TSS, total soluble solids; DB, double bulb formation; and TI, thrips incidence.

The analysis of variance (ANOVA) from the AMMI model indicated that the mean sum of squares was significant (*P* < 0.05) for environments, genotypes, genotype × environment (G × E) interaction effects, and all principal components (PCs) in the case of marketable yield, average bulb weight, and TSS. For double-bulb formation, all sources of variation were significant except replication and PC7. In contrast, for days to harvest, replication, PC5, PC6, and PC7 were non-significant, while all other sources showed significant effects. For thrips incidence, significant variation was observed for environments, genotypes, G × E interaction, PC1, and PC2 (**Table 6**). The AMMI1 biplot displays PC1 on the vertical axis, representing the extent of GEI (**Figure 9**). Genotypes or environments with PC1 scores near zero are considered less affected by interaction effects. For marketable yield, environments such as E4, E8, and E3; for days to harvest, E3 and E1; for average bulb weight, E8 and E6; for TSS, E1 and E3; for double-bulb formation, E8; and for thrips incidence, E7 and E1 had PC1 values close to zero, indicating minimal interaction. Similarly, genotypes showing stable performance with PC1 values near zero included G8 (RO-1657), G16 (RO-1758), and G13 (RO-1747) for marketable yield; G12 (RO-1741), G6 (RO-1642), and G7 (RO-1654) for days to harvest; G5 (RO-1625) and G18 (RO-1770) for average bulb weight; G21 (RO-1784), G15 (RO-1757), and G12 (RO-1741) for TSS; G11 (RO-1672), G5 (RO-1625), and G21 (RO-1784) for double-bulb formation; and G2 (RO-1620), G11 (RO-1672), and G24 (Bhima Shakti) for thrips incidence, suggesting limited sensitivity to environmental variability.

**Table 6:** Combined analysis of variance using AMMI biplot approach.

Source of variation	Df	MY (q/ha)		DTH (days)		ABW (g)		TSS (°Brix)		DB (%)		TI (scale 1-5)	
		MSS	Prop (%) (Accum.)	MSS	Prop (%) (Accum.)	MSS	Prop (%) (Accum.)	MSS	Prop (%) (Accum.)	MSS	Prop (%) (Accum.)	MSS	Prop (%) (Accum.)
ENV	7	823101.99*		2742.09*		3 3236.36*		180.82*		325.75*		81.87*	
REP(ENV)	16	80.87		4.86		2.73		0.09		0.00		0.57	
GEN	23	21767.49*		69.46*		488.54*		1.53*		6.08*		0.77*	
GEN: ENV	161	9367.33*		31.46*		213.81*		1.87*		10.09*		0.64*	
PC1	29	16069.66*	30.9	82.56*	47.3	538.92*	45.4	4.38*	42.2	38.54*	68.8	2.10*	59.1
PC2	27	14326.74*	25.6 (56.5)	48.78*	26 (73.3)	233.51*	18.3 (63.7)	2.39*	21.5 (63.7)	11.87*	19.7 (88.6)	0.87*	22.8 (81.9)
PC3	25	10755.00*	17.8 (74.4)	30.38*	15 (88.3)	212.94*	15.5 (79.2)	1.38*	11.5 (75.1)	3.80*	5.8 (94.4)	0.53	12.8 (94.7)
PC4	23	8376.80*	12.8 (87.2)	17.94*	8.1 (96.4)	131.68*	8.8 (88)	1.49*	11.4 (86.5)	2.46*	3.5 (97.9)	0.24	5.3 (100)
PC5	21	5185.93*	7.2 (94.4)	5.85	2.4 (98.9)	120.86*	7.4 (95.4)	1.11*	7.7 (94.2)	1.44*	1.9 (99.8)	0.00	0 (100)
PC6	19	2805.19*	3.5 (97.9)	3.06	1.1 (100)	51.65*	2.9 (98.2)	0.76*	4.8 (99)	0.21*	0.2 (100)	0.00	0 (100)
PC7	17	1856.11*	2.1 (100)	0.00	0 (100)	36.38*	1.8 (100)	0.17*	1 (100)	0.00	0 (100)	0.00	0 (100)
Error	368	50.80		8.50		3.21		0.08		0.01		0.44	
Total	736	12,634.02		46.37		426.58		2.63		7.70		1.32	

*Indicate significance at *P* < 0.05; Df, degree of freedom; MSS, mean sum of square; Prop (%) (Accum.), proportion of GEI explained by each principal component and its cumulative proportion; ENV, environment; REP, replication; GEN, genotype; GEN: ENV, genotype × environment interaction; PC, principal component; MY, marketable yield; DTH, days to harvest; ABW, average bulb weight; TSS, total soluble solids; DB, double bulb formation; and TI, thrips incidence

**Figure 10:**
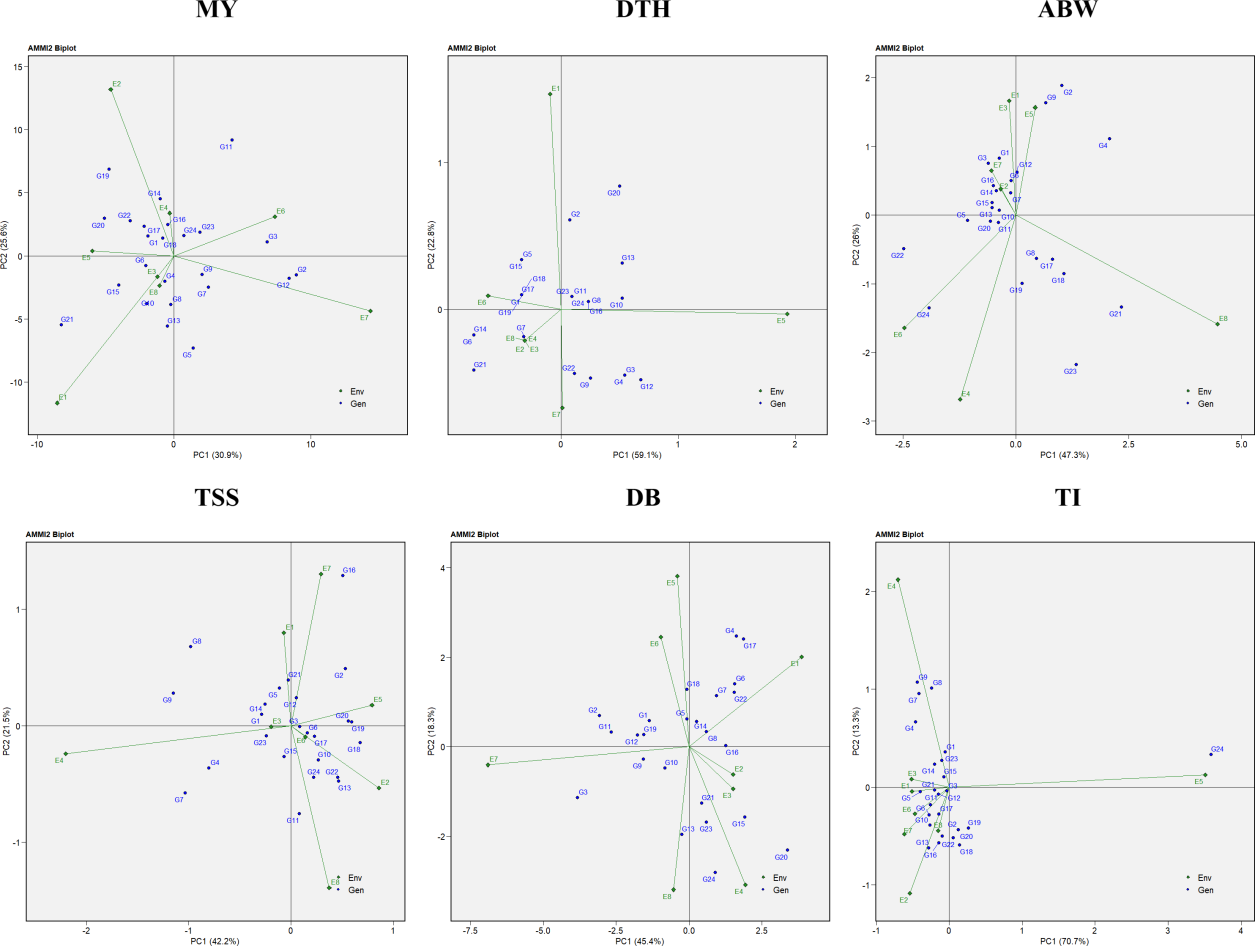
AMMI-2 biplot representation of onion genotypes across eight diverse environments for the evaluated traits: MY, marketable yield; DTH, days to harvest; ABW, average bulb weight; TSS, total soluble solids; DB, double bulb formation; and TI, thrips incidence.

The AMMI2 biplot, incorporating both the first and second principal components (PC1 and PC2), provided a comprehensive understanding of genotype stability and G×E interactions. In this study, PC1 and PC2 together explained 56.5% of the variation for marketable yield, 81.9% for days to harvest, 73.3% for average bulb weight, 63.7% for TSS, 63.7% for double-bulb formation, and 84.0% for thrips incidence (**Figure 10**). Genotypes located near the origin, where both PC1 and PC2 scores are close to zero, were considered more stable due to their minimal interaction with environmental variation.

Environments with shorter vector lengths, indicating weaker interactions, included E3, E8, and E4 for marketable yield; E2, E3, E4, and E8 for days to harvest; E2 and E7 for average bulb weight; E3 and E6 for TSS; E2 and E3 for double-bulb formation; and E1, E3, E6, and E8 for thrips incidence. Their proximity to the origin suggests they are suitable for identifying genotypes with consistent performance.

Similarly, stable genotypes located near the origin were G18 (RO-1770) and G24 (Bhima Shakti) for marketable yield; G11 (RO-1672), G23 (Bhima Kiran), and G24 (Bhima Shakti) for days to harvest; G10 (RO-1665) and G11 (RO-1672) for average bulb weight; G3 (RO-1621) and G6 (RO-1642) for TSS; G8 (RO-1657) and G14 (RO-1751) for double-bulb formation; and G3 (RO-1621) and G15 (RO-1757) for thrips incidence, highlighting their potential for use in future breeding programs targeting stable performance across diverse environments.

## 4 Discussion

Onion is predominantly an outcrossing species, which promotes extensive genetic variability among genotypes. This intrinsic variability underpins both the potential for rapid genetic gain through hybridization and the pronounced GEI commonly observed in multilocation trials. Stability and adaptability assessments help identify consistently performing genotypes across varying environmental conditions (Becker and Leon, 1988). Environmental variation across locations and years generates significant GEI, complicating selection and necessitating robust multi-environment analyses (Annicchiarico, 2002; Yan and Kang, 2003). Such GEI effects have been reported in onion and other vegetable crops (Cuartero and Cubero, 1982; Yildirim and Caliskan, 1985; Khar et al., 2007; Dia et al., 2018; Tahir et al., 2020; Tignegre et al., 2022; Gupta et al., 2024a). The identification of genotypes with both high mean performance and stability is therefore crucial for the development of cultivars suited to variable agro-climatic regions (Gupta et al., 2022). Onion genotypes are evaluated in multi-environment trials (METs) to identify suitable agro-climatic zones for the release and large-scale cultivation of varieties with consistent performance. Studies on GEI thus provide valuable preliminary insights for guiding breeding strategies.

Our study aimed to evaluate how the combination of BLUPs, GGE, and AMMI can identify stable and superior onion genotypes. Combined ANOVA for both GGE and AMMI showed significant GEI at *P* < 0.05 for all traits evaluated in the tested onion genotypes (**Tables 4**, **6**). This strengthened the predictive accuracy of BLUPs, GGE, and AMMI analyses and enabled the identification of genotypes with either broad or specific adaptability across environments (Akter et al., 2015). The AMMI model captures both additive and multiplicative components of variation, whereas the GGE biplot focuses on genotype and G × E effects, offering better visualization of performance and stability (Gauch, 2006; Yan and Tinker, 2006; Yan et al., 2007). Genotype performance was evaluated using BLUP-derived values rather than simple means, as BLUP provides more accurate and unbiased predictions by accounting for random effects and variance components (Nardino et al., 2016; Sood et al., 2020; Verma and Singh, 2021; Taleghani et al., 2023).

To assess stability, adaptability, and performance simultaneously, the harmonic mean of relative performance of genotypic values (HMRPGV) was employed (**Table 3**). Although HMGV and RPGV can be reported separately, HMRPGV alone was preferred as it integrates both measures into a single, robust index, offering a more reliable and concise criterion for genotype selection across environments (Rodovalho et al., 2015; Peixoto et al., 2021). These findings are in agreement with previous reports (Aina et al., 2009; Tahir et al., 2020; Gupta et al., 2024a, 2024b; Tiwari et al., 2025). The BLUP stability parameters were highly consistent with overall genotype rankings, indicating stable performance across traits and suggesting a strong association between BLUP estimates and yield. Genotypes G24 (Bhima Shakti), G23 (Bhima Kiran), G11 (RO-1672), G19 (RO-1773), and G17 (RO-1769) were identified as stable genotypes with high marketable yield. Similar patterns have been reported in other crops, including sunflower (Aboye and Edo, 2024), soybean (Freiria et al., 2018), finger millet (Anuradha et al., 2022), and lentil (Hossain et al., 2023).

Using GGE biplots (**Figure 2**), several genotypes—including G4 (RO-1622), G24 (Bhima Shakti), G13 (RO-1747), and G8 (RO-1657)—were identified as stable performers for marketable yield. Such stable performers not only hold promise for direct varietal release but also serve as valuable parental lines in hybrid and OPV breeding programs aimed at enhancing yield stability (John et al., 2025). The identification of stable genotypes has been reported in various crops such as wheat (Omrani et al., 2022), winter lentils (Hossain et al., 2023), sorghum (Wang et al., 2023), and tomato (Tiwari et al., 2025).

The which-won-where view of the GGE biplot (**Figure 3**) helps identify winning genotypes, the presence of crossovers, separate mega-environments, and genotypes adapted to specific locations (Yan et al., 2000). Three mega-environments were identified, with G11 (RO-1672) and G24 (Bhima Shakti) emerging as winning genotypes in four of the eight locations (E2, E3, E4, and E6) for marketable yield. Similar findings in onion and other crops have emphasized the role of mega-environment analysis in capturing environmental heterogeneity and guiding genotype recommendations (Erdemci, 2018; Daemo et al., 2023; Mitrovia et al., 2012; Rad et al., 2013; Vaezi et al., 2017). However, it is important to note that mega-environment patterns may vary across years due to seasonal and climatic fluctuations. The crossover interactions observed indicate that different genotypes perform best in different environments, leading to mega-environment formation. While the current analysis used data from two consecutive *rabi* seasons at the same eight locations, further validation over additional years would strengthen confidence in the stability and repeatability of these mega-environments for long-term genotype recommendations (Aboye and Edo, 2024).

The discriminativeness vs. representativeness view of the GGE biplot (**Figure 4**) identifies ideal test environments—those that effectively differentiate genotypes and represent the overall target environment. Environments E6 (ICAR–DOGR, Pune) and E4 (JNKVV, Jabalpur) were the most effective in discriminating among genotypes and representing overall test conditions for onion trait evaluation.

The ranking view of the GGE biplot (**Figure 5**) displays genotypes based on their mean performance and stability. Genotypes located closer to the ideal genotype position, represented at the center of the concentric circles, are considered both high performing and stable across environments (Yan and Kang, 2003). For yield performance, genotypes G24 (Bhima Shakti), followed by G11 (RO-1672), G23 (Bhima Kiran), G17 (RO-1769), and G19 (RO-1773), were recognized as the most promising (Enyew et al., 2021; Omrani et al., 2022).

Similarly, the ranking environment’s view in the GGE biplot (**Figure 6**) evaluates environments based on their mean performance and representativeness. Those closest to the ideal environment, located near the center of the concentric circles on the AEC axis, are considered most suitable for genotype evaluation (Yan et al., 2007). Based on the ranking view of the GGE biplot, the most suitable environments were E6>E2>E4 for marketable yield; E2> E8 >E4 for average bulb weight; and E4>E3> E6 for TSS. The influence of environments on genotype selection and the identification of ideal environments has also been reported in other studies (Esan et al., 2023; Tiwari et al., 2025).

Since the GT biplot was less effective for multi-trait selection, the GYT biplot was applied by integrating yield with each trait, thereby capturing positive associations between traits (**Figure 7**; Yan and Fregeau-Reid, 2018). To complement this, the MGIDI index ranked genotypes based on their distance from an ideal ideotype, enabling more accurate multi-trait selection (**Figure 8**; Olivoto and Nardino, 2021). Both approaches consistently identified G24 (Bhima Shakti), G11 (RO-1672), G23 (Bhima Kiran), and G19 (RO-1773) as stable, high-yielding genotypes, in agreement with earlier reports (Habib et al., 2024; Yue et al., 2022).

Moreover, the AMMI model was employed in this study to assess GEI and evaluate genotype stability (**Figures 9**, **10**). AMMI was used alongside GGE because, while GGE effectively visualizes which-won-where patterns and mega-environments, AMMI provides statistical partitioning of main and interaction effects, improving stability accuracy (Purchase, 1997; Yan and Kang, 2003; Gauch, 2013). Environments with shorter vectors and closer proximity to the origin are considered more reliable for selecting widely adaptable genotypes (Gauch, 2006). This study identified stable genotypes G18 (RO-1770) and G24 (Bhima Shakti), along with environments E8 (TNAU, Coimbatore) and E4 (JNKVV, Jabalpur), as the most suitable for yield. Similar use of AMMI-based stability analysis has been reported in groundnut and wheat (Kebede and Getahun, 2017; Omrani et al., 2022).

**Figure 11:**
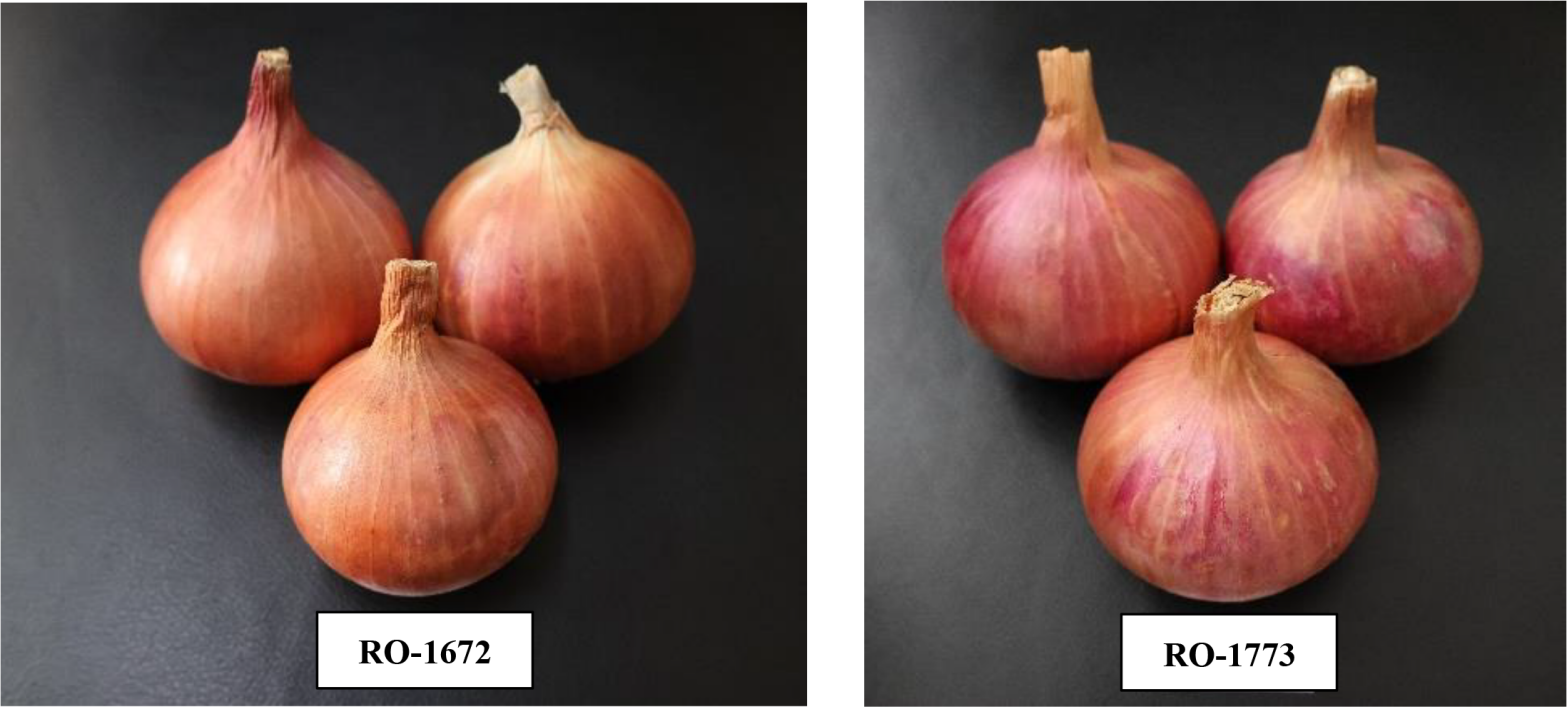
Promising onion genotypes exhibiting stable and superior performance across eight diverse test environments.

Based on the results of multivariate analyses using GGE, AMMI biplots, and BLUP, the genotypes Bhima Shakti (G24), RO-1672 (G11), Bhima Kiran (G23), and RO-1773 (G19) exhibited stable and superior performance across eight diverse testing environments (**Figure 11**). These analytical approaches effectively unraveled the complexity of genotype × environment interactions, aiding in the selection of widely adapted and desirable genotypes. Therefore, these genotypes are considered well suited for cultivation during the *rabi* season.

## 5 Conclusion

The combined use of BLUP, GGE biplot, AMMI, and multivariate selection indices (MGIDI and GYT) effectively distinguished yield potential, stability, and multi-trait performance among 24 red onion genotypes evaluated across eight *rabi* environments. For marketable yield, genotypes G24 (Bhima Shakti), G11 (RO-1672), G23 (Bhima Kiran), G17 (RO-1769), and G19 (RO-1773) were consistently superior. Early maturity was observed in G2 (RO-1620) and G4 (RO-1622), while higher average bulb weight was recorded in G24, G11, G19, and G23. Genotypes G7 (RO-1654), G4 (RO-1622), and G9 (RO-1664) performed best for TSS; G17 (RO-1769) and G18 (RO-1770) exhibited fewer double bulbs; and G6 (RO-1642), G14 (RO-1751), and G21 (RO-1784) showed reduced thrips incidence.

Environment E6 was identified as most suitable for yield and double-bulb traits, E4 for days to harvest and TSS, E8 for average bulb weight, and E5 for thrips resistance. The MGIDI ranking further confirmed G24 (Bhima Shakti), G11 (RO-1672), G23 (Bhima Kiran), and G19 (RO-1773) as the most promising multi-trait genotypes, holding strong potential to enhance onion productivity during the *rabi* season in India.

This study demonstrates that combining BLUP with graphical approaches (GGE/AMMI) and multi-trait indices (MGIDI/GYT) yields robust, actionable selection decisions for onion breeding. Based on these findings, Bhima Shakti, RO-1672, Bhima Kiran, and RO-1773 are recommended as priority candidates for advancement in breeding programs and large-scale evaluation under farmer field conditions. Future studies should focus on incorporating molecular approaches to dissect stability and multi-trait expression in these promising lines, thereby accelerating the development of stable, high-yielding onion cultivars suited to diverse *rabi* environments.

## Data Availability

The original contributions presented in the study are included in the article/**Supplementary Material**. Further inquiries can be directed to the corresponding author.
