# Supplementary material for: Stability and adaptability assessment of red onion genotypes using AMMI, GGE, BLUP, and multivariate indices
Source: Front Plant Sci. 2025 Oct 22;16:1694946. doi: 10.3389/fpls.2025.1694946 (PMC12586005; doi:10.3389/fpls.2025.1694946)
Supplement: Supplementary file 1 [file Table1.docx]

**Supplementary File 1**

**Title: Stability and Adaptability Assessment of Red Onion Genotypes Using AMMI, GGE, BLUP, and Multivariate Indices**

Seasonal weather parameters recorded across the eight agro-climatic test environments (E1-E8).

| **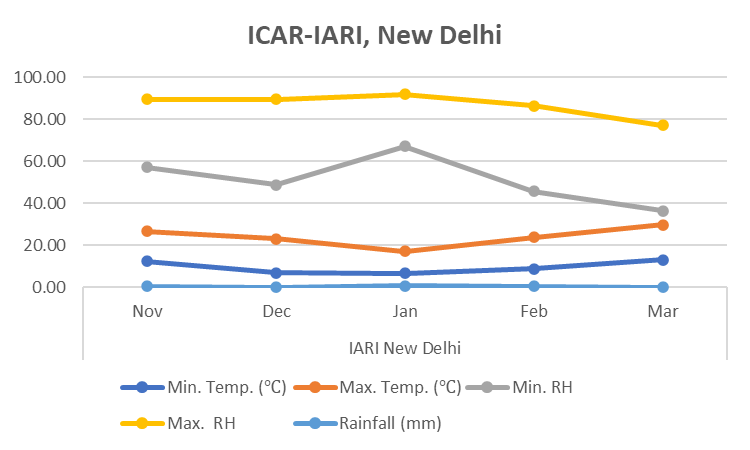** | **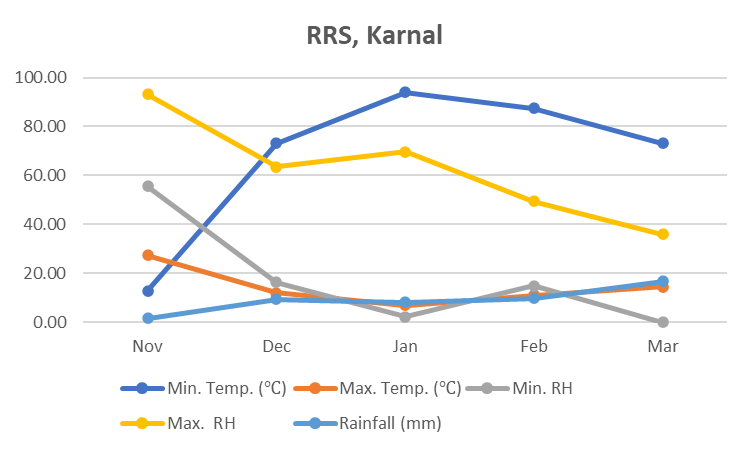** |
| --- | --- |
| 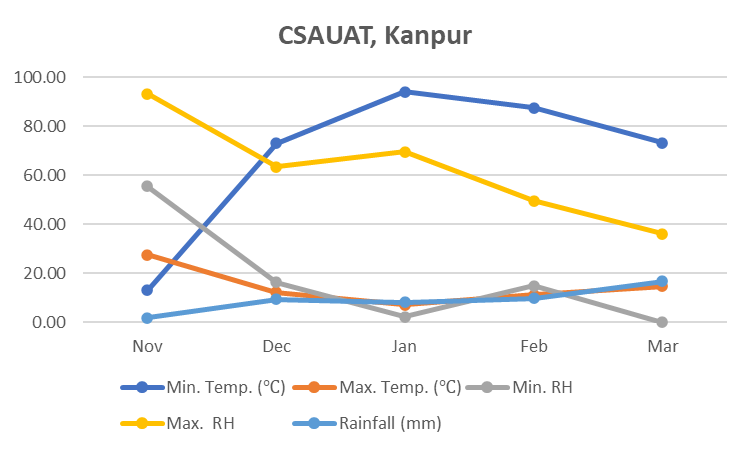 | 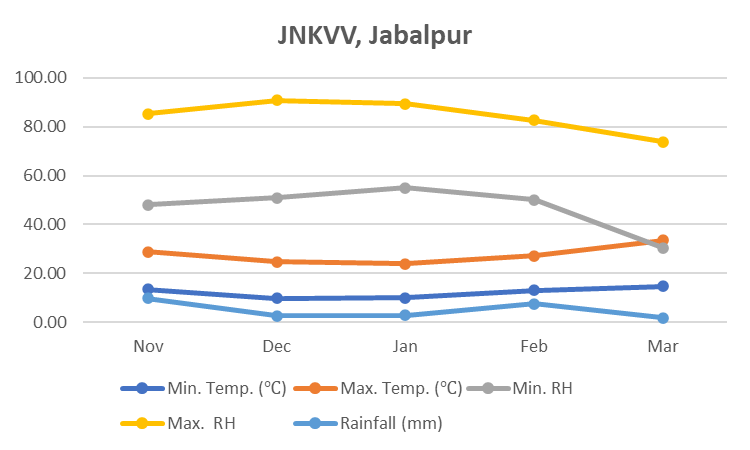 |
| **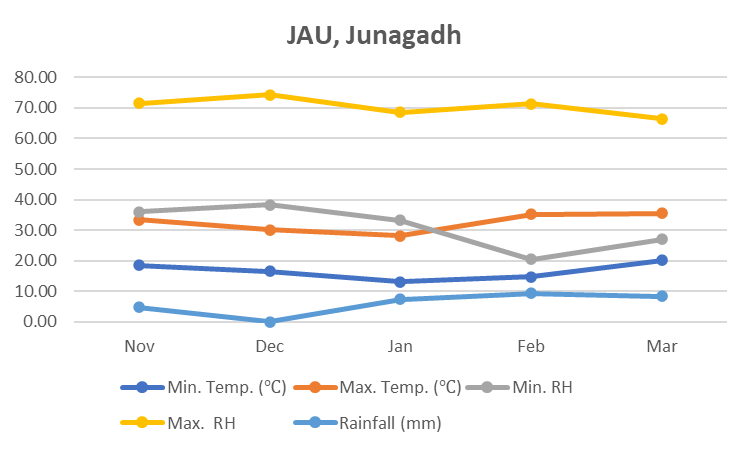** | **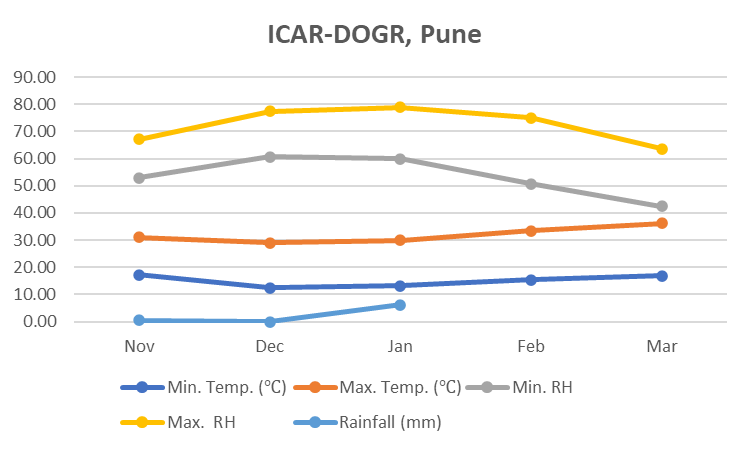** |
| **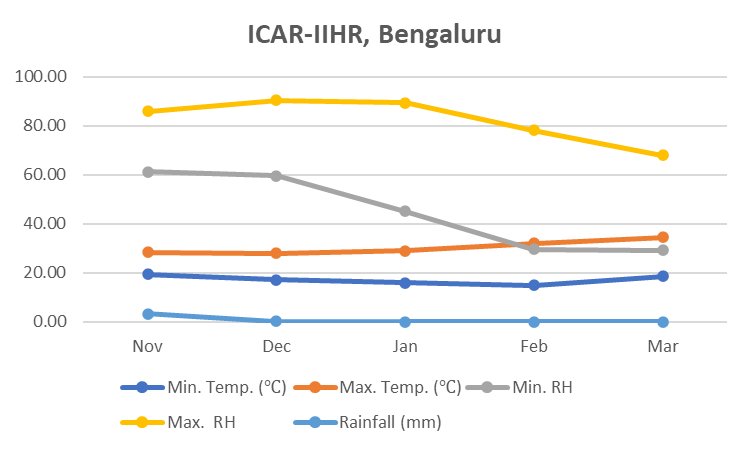** | **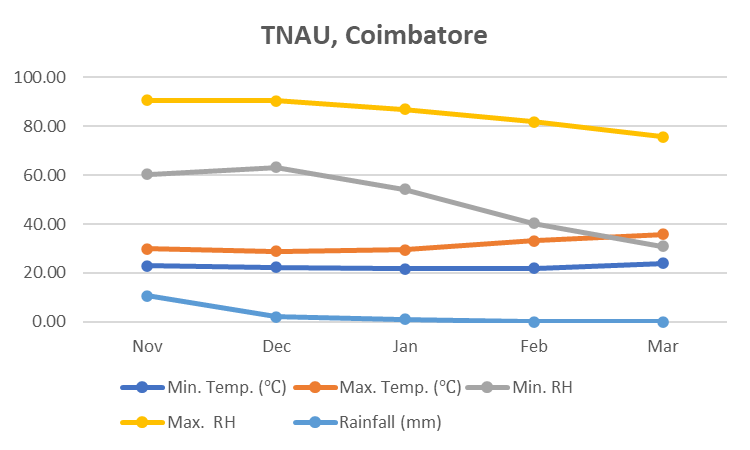** |
