# Supplementary material for: Stability and adaptability assessment of red onion genotypes using AMMI, GGE, BLUP, and multivariate indices
Source: Front Plant Sci. 2025 Oct 22;16:1694946. doi: 10.3389/fpls.2025.1694946 (PMC12586005; doi:10.3389/fpls.2025.1694946)
Supplement: Supplementary file 2 [file Table2.doc]

**Supplementary File 2**

**Title: Stability and Adaptability Assessment of Red Onion Genotypes Using AMMI, GGE, BLUP, and Multivariate Indices**

Performance averages of onion genotypes across eight diverse test environments

| **Code** | **Genotypes** | **Marketable yield (q/ha)** | | | | | | | | | **Days to harvest** | | | | | | | | | **Average bulb weight (g)** | | | | | | | | |
| --- | --- | --- | --- | --- | --- | --- | --- | --- | --- | --- | --- | --- | --- | --- | --- | --- | --- | --- | --- | --- | --- | --- | --- | --- | --- | --- | --- | --- |
|  |  | **E1** | **E2** | **E3** | **E4** | **E5** | **E6** | **E7** | **E8** | **Mean** | **E1** | **E2** | **E3** | **E4** | **E5** | **E6** | **E7** | **E8** | **Mean** | **E1** | **E2** | **E3** | **E4** | **E5** | **E6** | **E7** | **E8** | **Mean** |
| G1 | RO-1619 | 241.18 | 211.33 | 212.50 | 211.00 | 312.22 | 492.13 | 244.40 | 177.60 | 262.80 | 126.00 | 112.00 | 130.00 | 111.00 | 131.00 | 119.33 | 116.33 | 123.67 | 121.17 | 59.60 | 46.40 | 51.06 | 41.98 | 77.50 | 83.78 | 113.72 | 50.70 | 65.59 |
| G2 | RO-1620 | 141.18 | 110.00 | 201.75 | 171.79 | 325.00 | 516.48 | 442.10 | 193.20 | 262.69 | 126.00 | 112.00 | 130.00 | 107.33 | 133.00 | 111.00 | 116.67 | 126.67 | 120.33 | 62.00 | 36.73 | 50.71 | 45.12 | 80.35 | 94.46 | 129.42 | 59.10 | 69.74 |
| G3 | RO-1621 | 80.00 | 75.23 | 119.00 | 220.00 | 312.22 | 488.00 | 338.50 | 182.70 | 226.96 | 126.00 | 116.00 | 130.00 | 113.00 | 134.00 | 121.00 | 116.67 | 123.67 | 122.54 | 40.45 | 28.21 | 41.97 | 41.79 | 61.30 | 90.39 | 122.53 | 54.30 | 60.12 |
| G4 | RO-1622 | 227.94 | 106.67 | 151.00 | 178.33 | 275.56 | 451.97 | 235.00 | 159.30 | 223.22 | 126.00 | 112.00 | 130.00 | 108.00 | 131.00 | 111.00 | 116.33 | 133.67 | 121.00 | 82.35 | 40.31 | 42.32 | 36.43 | 66.30 | 84.85 | 92.03 | 37.25 | 60.23 |
| G5 | RO-1625 | 311.77 | 80.78 | 178.50 | 171.33 | 405.56 | 481.33 | 375.90 | 170.30 | 271.93 | 126.00 | 116.00 | 130.00 | 117.00 | 130.00 | 123.00 | 122.00 | 124.00 | 123.50 | 70.85 | 30.29 | 46.91 | 42.22 | 69.80 | 82.77 | 102.60 | 52.75 | 62.27 |
| G6 | RO-1642 | 203.21 | 105.78 | 184.00 | 221.67 | 387.78 | 469.37 | 225.60 | 183.60 | 247.63 | 126.00 | 118.00 | 130.00 | 113.33 | 135.00 | 121.00 | 113.67 | 126.67 | 122.96 | 69.20 | 36.62 | 46.13 | 42.81 | 75.90 | 85.32 | 85.44 | 50.35 | 61.47 |
| G7 | RO-1654 | 239.22 | 107.56 | 197.00 | 245.00 | 394.44 | 534.51 | 342.50 | 169.00 | 278.65 | 126.00 | 120.00 | 130.00 | 115.00 | 133.00 | 120.00 | 117.67 | 127.33 | 123.63 | 75.10 | 38.79 | 51.33 | 46.34 | 71.30 | 83.85 | 98.77 | 44.15 | 63.70 |
| G8 | RO-1657 | 288.24 | 119.78 | 219.85 | 173.67 | 385.00 | 531.65 | 298.20 | 164.90 | 272.66 | 126.00 | 118.00 | 130.00 | 118.67 | 132.00 | 121.00 | 122.00 | 133.00 | 125.08 | 72.50 | 39.92 | 52.43 | 52.18 | 68.80 | 89.45 | 102.03 | 48.70 | 65.75 |
| G9 | RO-1664 | 176.47 | 79.22 | 97.00 | 216.79 | 327.78 | 461.49 | 286.00 | 196.30 | 230.13 | 126.00 | 118.00 | 130.00 | 111.00 | 132.00 | 111.00 | 122.67 | 126.67 | 122.17 | 67.00 | 28.57 | 43.19 | 42.29 | 68.15 | 83.03 | 114.29 | 61.35 | 63.48 |
| G10 | RO-1665 | 229.41 | 111.22 | 99.50 | 148.67 | 362.22 | 334.32 | 294.40 | 185.20 | 220.62 | 126.00 | 114.00 | 130.00 | 116.00 | 131.00 | 121.00 | 123.67 | 127.00 | 123.58 | 67.75 | 32.34 | 48.44 | 44.04 | 63.30 | 76.12 | 111.55 | 49.95 | 61.69 |
| G11 | RO-1672 | 112.55 | 330.56 | 144.70 | 263.33 | 422.22 | 512.19 | 406.50 | 184.60 | 297.08 | 126.00 | 117.00 | 130.00 | 116.67 | 133.00 | 123.00 | 121.67 | 128.00 | 124.42 | 63.55 | 59.50 | 45.17 | 47.52 | 81.30 | 92.11 | 130.73 | 59.40 | 72.41 |
| G12 | RO-1741 | 142.94 | 101.56 | 152.50 | 233.67 | 333.33 | 446.38 | 461.30 | 171.80 | 255.44 | 126.00 | 116.00 | 130.00 | 113.00 | 133.00 | 120.00 | 123.00 | 128.00 | 123.63 | 57.55 | 38.08 | 42.66 | 40.32 | 68.15 | 78.37 | 114.48 | 44.25 | 60.48 |
| G13 | RO-1747 | 311.77 | 121.48 | 120.10 | 177.00 | 252.78 | 395.49 | 312.50 | 162.60 | 231.72 | 126.00 | 115.00 | 130.00 | 117.00 | 132.00 | 121.00 | 124.33 | 126.33 | 123.96 | 54.20 | 37.74 | 54.19 | 54.21 | 56.35 | 76.28 | 104.13 | 43.00 | 60.01 |
| G14 | RO-1751 | 158.34 | 209.56 | 189.00 | 244.67 | 295.56 | 451.33 | 231.30 | 160.50 | 242.53 | 126.00 | 115.00 | 130.00 | 113.67 | 131.00 | 122.00 | 124.33 | 126.67 | 123.58 | 62.65 | 48.38 | 43.69 | 44.22 | 60.10 | 82.70 | 100.49 | 32.60 | 59.35 |
| G15 | RO-1757 | 235.30 | 124.45 | 203.15 | 217.67 | 422.22 | 384.63 | 255.40 | 181.80 | 253.08 | 126.00 | 115.00 | 130.00 | 115.00 | 130.00 | 121.00 | 121.67 | 125.67 | 123.04 | 63.15 | 36.17 | 51.27 | 44.95 | 47.75 | 78.54 | 82.90 | 47.30 | 56.50 |
| G16 | RO-1758 | 172.27 | 179.78 | 131.50 | 213.42 | 375.00 | 465.56 | 267.60 | 196.70 | 250.23 | 126.00 | 117.00 | 130.00 | 113.00 | 129.00 | 121.00 | 122.67 | 125.67 | 123.04 | 65.50 | 46.26 | 44.58 | 46.55 | 70.00 | 81.46 | 89.99 | 52.25 | 62.07 |
| G17 | RO-1769 | 249.58 | 228.23 | 159.55 | 231.08 | 450.00 | 516.57 | 286.50 | 155.40 | 284.61 | 126.00 | 119.00 | 130.00 | 119.67 | 133.00 | 119.33 | 122.67 | 134.67 | 125.54 | 78.00 | 43.71 | 49.38 | 47.24 | 80.25 | 86.95 | 90.83 | 40.60 | 64.62 |
| G18 | RO-1770 | 254.70 | 214.78 | 115.50 | 248.96 | 391.67 | 483.62 | 312.10 | 153.50 | 271.85 | 126.00 | 118.00 | 130.00 | 119.67 | 135.00 | 121.00 | 120.67 | 136.67 | 125.88 | 78.05 | 47.18 | 45.60 | 47.24 | 71.45 | 84.53 | 111.89 | 42.15 | 66.01 |
| G19 | RO-1773 | 229.41 | 316.67 | 192.80 | 209.97 | 438.89 | 544.95 | 217.10 | 171.20 | 290.12 | 126.00 | 119.00 | 130.00 | 122.00 | 132.00 | 122.00 | 126.00 | 133.00 | 126.25 | 65.60 | 55.90 | 54.37 | 47.32 | 63.25 | 97.50 | 122.80 | 43.30 | 68.76 |
| G20 | RO-1783 | 156.87 | 183.78 | 126.50 | 230.28 | 363.39 | 322.60 | 199.80 | 181.80 | 220.63 | 126.00 | 118.00 | 130.00 | 119.00 | 131.00 | 119.33 | 121.00 | 125.67 | 123.75 | 67.70 | 47.30 | 50.47 | 51.16 | 52.85 | 66.39 | 75.23 | 50.60 | 57.71 |
| G21 | RO-1784 | 308.83 | 125.45 | 136.00 | 194.42 | 442.94 | 276.51 | 243.30 | 201.30 | 241.09 | 126.00 | 119.00 | 130.00 | 122.67 | 132.00 | 114.67 | 122.67 | 142.67 | 126.21 | 65.70 | 38.98 | 52.26 | 50.97 | 71.85 | 79.84 | 99.18 | 65.45 | 65.53 |
| G22 | RO-1824 | 103.83 | 122.12 | 168.00 | 215.37 | 373.78 | 363.75 | 185.10 | 158.70 | 211.33 | 126.00 | 117.00 | 130.00 | 123.67 | 129.00 | 123.33 | 122.67 | 117.33 | 123.63 | 59.40 | 35.48 | 50.69 | 47.80 | 71.50 | 89.09 | 82.74 | 38.95 | 59.46 |
| G23 | Bhima Kiran (C) | 225.49 | 260.78 | 199.50 | 288.17 | 400.00 | 425.52 | 427.60 | 190.80 | 302.23 | 126.00 | 116.00 | 130.00 | 119.67 | 133.00 | 129.00 | 119.00 | 142.67 | 126.92 | 67.65 | 51.02 | 48.43 | 55.91 | 71.90 | 79.94 | 101.67 | 66.50 | 67.88 |
| G24 | Bhima Shakti (C) | 322.80 | 326.00 | 178.65 | 302.05 | 395.17 | 481.97 | 442.60 | 193.50 | 330.34 | 126.00 | 119.00 | 130.00 | 124.33 | 132.00 | 129.00 | 119.00 | 123.67 | 125.37 | 83.55 | 57.58 | 55.47 | 74.46 | 62.05 | 83.23 | 116.27 | 60.30 | 74.11 |
| Mean | | 213.47 | 164.70 | 161.56 | 217.85 | 368.53 | 451.35 | 305.47 | 176.93 | 257.48 | 126.00 | 116.50 | 130.00 | 116.22 | 131.96 | 120.21 | 120.79 | 128.71 | 123.80 | 66.63 | 41.73 | 48.45 | 47.29 | 67.98 | 83.79 | 103.99 | 49.80 | 63.71 |
| C.D. (*P*<0.05) | | 8.83 | 7.91 | 8.01 | 8.68 | 16.83 | 17.62 | 12.59 | 8.11 |  | 4.91 | 4.81 | 5.03 | 4.02 | 4.48 | 4.66 | 5.44 | 4.86 |  | 3.34 | 1.91 | 2.23 | 1.97 | 2.96 | 3.63 | 4.13 | 2.61 |  |
| SE(m) | | 3.10 | 2.78 | 2.82 | 3.05 | 5.91 | 6.19 | 4.42 | 2.85 |  | 1.72 | 1.69 | 1.77 | 1.41 | 1.57 | 1.64 | 1.91 | 1.71 |  | 1.17 | 0.67 | 0.78 | 0.69 | 1.04 | 1.28 | 1.45 | 0.92 |  |
| SE(d) | | 4.39 | 3.93 | 3.98 | 4.31 | 8.36 | 8.75 | 6.25 | 4.03 |  | 2.44 | 2.39 | 2.50 | 2.00 | 2.23 | 2.31 | 2.70 | 2.41 |  | 1.66 | 0.95 | 1.11 | 0.98 | 1.47 | 1.80 | 2.05 | 1.30 |  |
| C.V. (%) | | 2.52 | 2.92 | 3.02 | 2.42 | 2.78 | 2.37 | 2.51 | 2.79 |  | 2.37 | 2.51 | 2.35 | 2.11 | 2.07 | 2.36 | 2.74 | 2.30 |  | 3.05 | 2.78 | 2.80 | 2.54 | 2.65 | 2.64 | 2.42 | 3.19 |  |

E1: ICAR-IARI, New Delhi; E2: RRS, Karnal; E3: CSAUAT, Kanpur; E4: JNKVV, Jabalpur; E5: JAU, Junagadh; E6: ICAR-DOGR, Pune; E7: ICAR-IIHR, Bengaluru; E8: TNAU, Coimbatore.

*Contd. Supplementary file 2*

| **Code** | **Genotypes** | **TSS (%)** | | | | | | | | | **Double bulbs (%)** | | | | | | | | | **Thrips incidence (scale 1-5)** | | | | | | | | |
| --- | --- | --- | --- | --- | --- | --- | --- | --- | --- | --- | --- | --- | --- | --- | --- | --- | --- | --- | --- | --- | --- | --- | --- | --- | --- | --- | --- | --- |
|  |  | **E1** | **E2** | **E3** | **E4** | **E5** | **E6** | **E7** | **E8** | **Mean** | **E1** | **E2** | **E3** | **E4** | **E5** | **E6** | **E7** | **E8** | **Mean** | **E1** | **E2** | **E3** | **E4** | **E5** | **E6** | **E7** | **E8** | **Mean** |
| G1 | RO-1619 | 9.87 | 12.22 | 13.26 | 10.20 | 13.68 | 12.00 | 11.37 | 12.65 | 11.91 | 0.00 | 0.00 | 0.00 | 0.00 | 0.00 | 1.01 | 8.00 | 0.00 | 1.13 | 3.00 | 1.00 | 1.00 | 1.00 | 3.00 | 2.00 | 1.00 | 1.00 | 1.63 |
| G2 | RO-1620 | 9.27 | 13.30 | 13.54 | 8.20 | 14.13 | 11.83 | 11.80 | 11.40 | 11.68 | 0.00 | 0.00 | 2.17 | 0.00 | 0.00 | 2.46 | 8.67 | 0.00 | 1.66 | 4.00 | 1.00 | 1.00 | 1.00 | 4.00 | 2.00 | 1.00 | 1.00 | 1.88 |
| G3 | RO-1621 | 8.80 | 12.77 | 13.20 | 9.20 | 13.45 | 11.76 | 11.33 | 12.30 | 11.60 | 0.00 | 0.00 | 0.00 | 1.11 | 0.00 | 1.21 | 6.33 | 0.00 | 1.08 | 2.00 | 1.00 | 1.00 | 1.00 | 5.00 | 2.00 | 1.00 | 1.00 | 1.75 |
| G4 | RO-1622 | 9.70 | 13.60 | 13.48 | 11.60 | 12.35 | 11.84 | 10.50 | 12.35 | 11.93 | 0.00 | 0.00 | 0.00 | 0.00 | 0.00 | 9.17 | 6.67 | 0.00 | 1.98 | 2.00 | 1.00 | 1.00 | 1.00 | 5.00 | 2.00 | 1.00 | 1.00 | 1.75 |
| G5 | RO-1625 | 9.54 | 12.90 | 13.00 | 9.20 | 12.03 | 11.53 | 11.47 | 11.55 | 11.40 | 0.00 | 0.00 | 2.19 | 1.63 | 1.50 | 2.65 | 3.00 | 0.00 | 1.37 | 3.00 | 1.00 | 1.00 | 1.00 | 3.00 | 2.00 | 0.00 | 1.00 | 1.50 |
| G6 | RO-1642 | 9.44 | 14.70 | 12.99 | 9.73 | 13.05 | 11.71 | 12.00 | 12.60 | 12.03 | 0.00 | 0.00 | 0.00 | 0.00 | 0.00 | 2.53 | 6.67 | 0.00 | 1.15 | 2.00 | 1.00 | 1.00 | 1.00 | 2.00 | 2.00 | 0.00 | 1.00 | 1.25 |
| G7 | RO-1654 | 9.54 | 13.15 | 13.95 | 12.27 | 12.68 | 12.20 | 10.30 | 12.85 | 12.12 | 0.00 | 0.00 | 0.00 | 0.00 | 0.00 | 0.54 | 5.67 | 0.00 | 0.78 | 2.00 | 1.00 | 1.00 | 1.00 | 3.00 | 2.00 | 0.00 | 1.00 | 1.38 |
| G8 | RO-1657 | 10.80 | 12.80 | 13.46 | 11.93 | 13.38 | 11.77 | 11.20 | 10.55 | 11.99 | 0.00 | 0.00 | 0.00 | 0.00 | 0.00 | 1.27 | 6.67 | 0.00 | 0.99 | 3.00 | 1.00 | 1.00 | 1.00 | 4.00 | 1.00 | 1.00 | 1.00 | 1.63 |
| G9 | RO-1664 | 9.50 | 12.35 | 13.69 | 12.20 | 12.53 | 11.83 | 11.63 | 11.55 | 11.91 | 0.00 | 0.00 | 0.00 | 0.00 | 0.00 | 2.13 | 10.00 | 0.00 | 1.52 | 2.00 | 1.00 | 1.00 | 1.00 | 4.00 | 1.00 | 1.00 | 1.00 | 1.50 |
| G10 | RO-1665 | 9.10 | 13.80 | 13.02 | 8.80 | 12.61 | 12.19 | 10.93 | 12.60 | 11.63 | 0.00 | 0.00 | 2.08 | 0.00 | 0.00 | 8.05 | 6.33 | 0.00 | 2.06 | 3.00 | 1.00 | 1.00 | 1.00 | 5.00 | 2.00 | 1.00 | 1.00 | 1.88 |
| G11 | RO-1672 | 9.37 | 13.75 | 13.49 | 9.60 | 12.90 | 11.92 | 10.80 | 14.10 | 11.99 | 0.00 | 0.00 | 0.00 | 0.00 | 0.00 | 3.57 | 6.33 | 0.00 | 1.24 | 3.00 | 1.00 | 1.00 | 1.00 | 4.00 | 2.00 | 1.00 | 1.00 | 1.75 |
| G12 | RO-1741 | 9.87 | 13.00 | 13.58 | 8.73 | 12.87 | 11.76 | 10.20 | 10.95 | 11.37 | 0.00 | 0.00 | 0.00 | 2.36 | 0.00 | 1.65 | 7.67 | 0.00 | 1.46 | 2.00 | 1.00 | 1.00 | 1.00 | 5.00 | 1.00 | 1.00 | 1.00 | 1.63 |
| G13 | RO-1747 | 8.84 | 13.90 | 13.94 | 8.00 | 12.35 | 12.03 | 10.37 | 12.60 | 11.50 | 0.00 | 0.00 | 0.00 | 0.00 | 0.00 | 1.56 | 7.67 | 0.00 | 1.15 | 3.00 | 1.00 | 1.00 | 1.00 | 5.00 | 2.00 | 0.00 | 1.00 | 1.75 |
| G14 | RO-1751 | 10.04 | 12.35 | 14.05 | 9.47 | 12.90 | 12.23 | 10.33 | 11.45 | 11.60 | 0.00 | 4.66 | 0.00 | 1.25 | 0.50 | 1.26 | 8.00 | 0.00 | 1.96 | 2.00 | 1.00 | 1.00 | 1.00 | 2.00 | 2.00 | 0.00 | 1.00 | 1.25 |
| G15 | RO-1757 | 9.47 | 13.50 | 13.90 | 9.80 | 13.57 | 11.71 | 10.50 | 12.40 | 11.86 | 0.00 | 0.00 | 0.00 | 2.22 | 0.00 | 10.75 | 7.67 | 0.00 | 2.58 | 3.00 | 1.00 | 1.00 | 1.00 | 3.00 | 2.00 | 0.00 | 1.00 | 1.50 |
| G16 | RO-1758 | 11.30 | 13.30 | 13.20 | 8.20 | 13.55 | 11.93 | 13.27 | 11.20 | 11.99 | 0.00 | 0.00 | 2.11 | 1.25 | 0.00 | 9.59 | 2.33 | 0.00 | 1.91 | 3.00 | 1.00 | 1.00 | 1.00 | 4.00 | 1.00 | 1.00 | 1.00 | 1.63 |
| G17 | RO-1769 | 9.70 | 13.50 | 12.56 | 9.00 | 13.10 | 11.69 | 11.20 | 12.70 | 11.68 | 0.00 | 0.00 | 2.59 | 2.63 | 0.00 | 0.34 | 3.67 | 0.00 | 1.15 | 3.00 | 1.00 | 1.00 | 1.00 | 3.00 | 2.00 | 1.00 | 1.00 | 1.63 |
| G18 | RO-1770 | 9.67 | 14.60 | 12.66 | 8.60 | 14.38 | 11.77 | 11.30 | 12.75 | 11.97 | 0.00 | 0.00 | 0.00 | 1.58 | 0.00 | 1.07 | 2.67 | 0.00 | 0.67 | 3.00 | 1.00 | 1.00 | 1.00 | 3.00 | 2.00 | 1.00 | 1.00 | 1.63 |
| G19 | RO-1773 | 9.60 | 13.20 | 13.46 | 8.20 | 14.61 | 11.88 | 10.93 | 12.40 | 11.79 | 0.00 | 0.00 | 0.00 | 1.14 | 0.00 | 6.04 | 2.33 | 0.00 | 1.19 | 3.00 | 1.00 | 1.00 | 1.00 | 3.00 | 2.00 | 1.00 | 1.00 | 1.63 |
| G20 | RO-1783 | 8.97 | 15.10 | 13.61 | 8.60 | 14.02 | 12.05 | 10.80 | 11.00 | 11.77 | 0.00 | 0.00 | 0.00 | 1.63 | 0.00 | 14.82 | 2.33 | 0.00 | 2.35 | 4.00 | 1.00 | 1.00 | 1.00 | 5.00 | 2.00 | 0.00 | 1.00 | 1.88 |
| G21 | RO-1784 | 10.07 | 13.50 | 13.47 | 10.20 | 13.66 | 11.76 | 13.23 | 13.05 | 12.37 | 0.00 | 0.00 | 0.00 | 0.00 | 0.00 | 2.90 | 2.00 | 0.00 | 0.61 | 2.00 | 1.00 | 1.00 | 1.00 | 2.00 | 2.00 | 1.00 | 1.00 | 1.38 |
| G22 | RO-1824 | 10.00 | 14.40 | 12.53 | 8.60 | 13.23 | 12.08 | 10.07 | 12.70 | 11.70 | 0.00 | 0.00 | 0.00 | 0.00 | 0.00 | 7.93 | 5.00 | 0.00 | 1.62 | 2.00 | 1.00 | 1.00 | 1.00 | 4.00 | 2.00 | 1.00 | 1.00 | 1.63 |
| G23 | Bhima Kiran (C) | 9.67 | 14.20 | 13.61 | 10.80 | 13.86 | 11.40 | 11.37 | 12.35 | 12.16 | 0.00 | 3.78 | 0.00 | 1.25 | 0.00 | 0.00 | 5.00 | 0.00 | 1.25 | 3.00 | 1.00 | 1.00 | 1.00 | 4.00 | 2.00 | 1.00 | 1.00 | 1.75 |
| G24 | Bhima Shakti (C) | 9.64 | 13.10 | 13.39 | 9.73 | 15.40 | 11.99 | 10.63 | 13.60 | 12.19 | 0.00 | 3.51 | 2.74 | 0.00 | 0.00 | 0.00 | 5.00 | 0.00 | 1.41 | 3.00 | 1.00 | 1.00 | 1.00 | 4.00 | 2.00 | 1.00 | 1.00 | 1.75 |
| Mean | | 9.66 | 13.46 | 13.38 | 9.62 | 13.35 | 11.87 | 11.15 | 12.24 | 11.84 | 0.00 | 0.50 | 0.58 | 0.75 | 0.08 | 3.85 | 5.65 | 0.00 | 1.43 | 2.71 | 1.00 | 1.00 | 1.00 | 3.71 | 1.83 | 0.71 | 1.00 | 1.62 |
| C.D. (*P*<0.05) | | 0.36 | 0.60 | 0.55 | 0.41 | 0.51 | 0.41 | 0.49 | 0.43 |  | _ | 0.08 | 0.06 | 0.05 | 0.02 | 0.24 | 0.30 | _ |  | 1.36 | 1.08 | 1.08 | 1.02 | 0.95 | 1.28 | 0.95 | 0.96 |  |
| SE(m) | | 0.13 | 0.21 | 0.19 | 0.15 | 0.18 | 0.14 | 0.17 | 0.15 |  | _ | 0.03 | 0.02 | 0.02 | 0.01 | 0.08 | 0.10 | _ |  | 0.48 | 0.38 | 0.38 | 0.36 | 0.33 | 0.45 | 0.33 | 0.34 |  |
| SE(d) | | 0.18 | 0.30 | 0.27 | 0.21 | 0.25 | 0.20 | 0.24 | 0.21 |  | _ | 0.04 | 0.03 | 0.03 | 0.01 | 0.12 | 0.15 | _ |  | 0.67 | 0.54 | 0.54 | 0.51 | 0.47 | 0.64 | 0.47 | 0.48 |  |
| C.V. (%) | | 2.25 | 2.70 | 2.50 | 2.62 | 2.34 | 2.10 | 2.65 | 2.13 |  | _ | 9.17 | 6.33 | 4.23 | 11.42 | 3.75 | 3.19 | _ |  | 30.51 | 65.52 | 65.52 | 61.97 | 15.53 | 42.49 | 81.73 | 58.51 |  |

E1: ICAR-IARI, New Delhi; E2: RRS, Karnal; E3: CSAUAT, Kanpur; E4: JNKVV, Jabalpur; E5: JAU, Junagadh; E6: ICAR-DOGR, Pune; E7: ICAR-IIHR, Bengaluru; E8: TNAU, Coimbatore.
