# Supplementary material for: Stability and adaptability assessment of red onion genotypes using AMMI, GGE, BLUP, and multivariate indices
Source: Front Plant Sci. 2025 Oct 22;16:1694946. doi: 10.3389/fpls.2025.1694946 (PMC12586005; doi:10.3389/fpls.2025.1694946)
Supplement: Supplementary file 3 [file Table3.doc]

**Supplementary File 3**

**Title: Stability and Adaptability Assessment of Red Onion Genotypes Using AMMI, GGE, BLUP, and Multivariate Indices**

Heatmap visualization of genotype performance across eight test environments

| **MY** | **DTH** | **ABW** |
| --- | --- | --- |
| 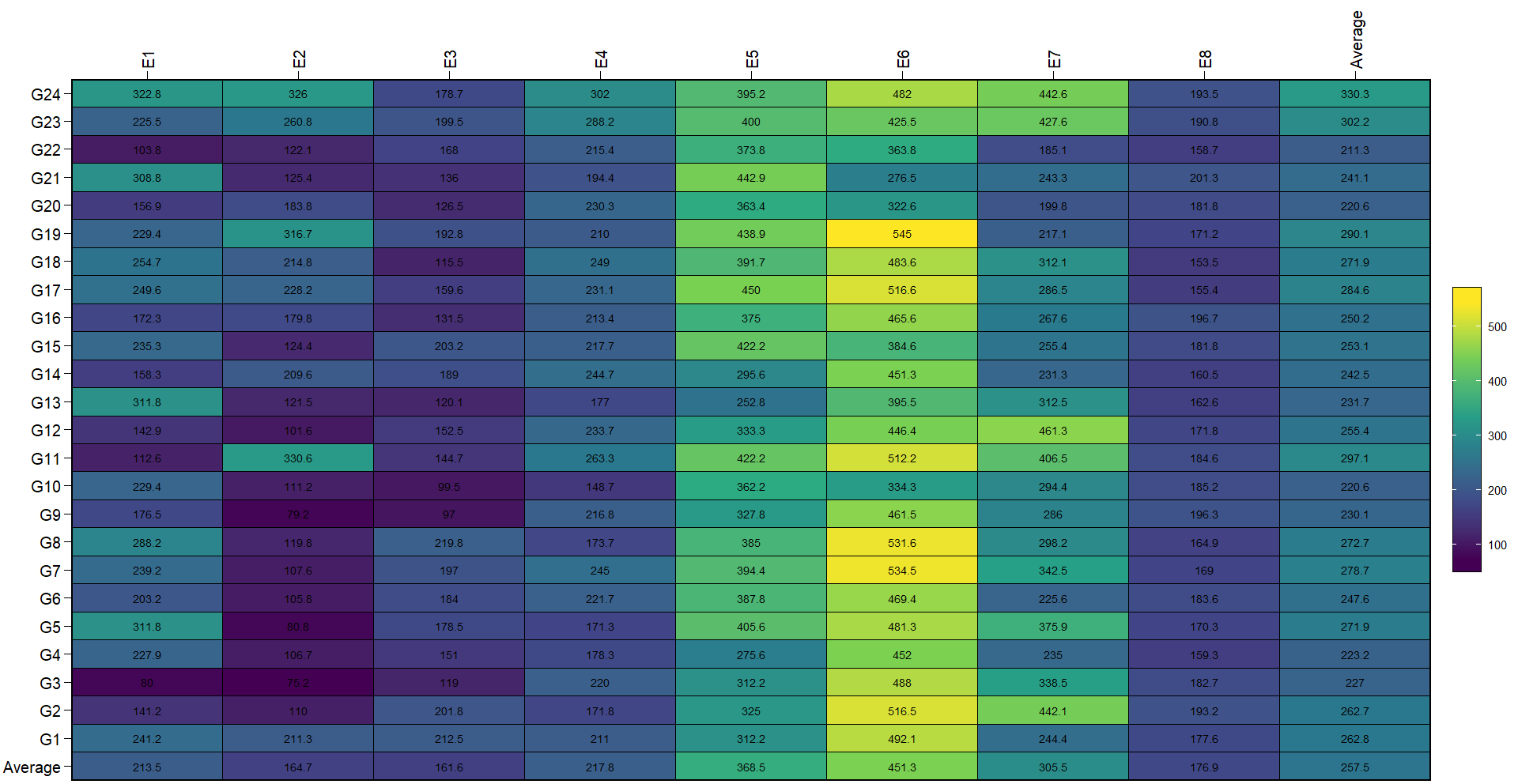 | 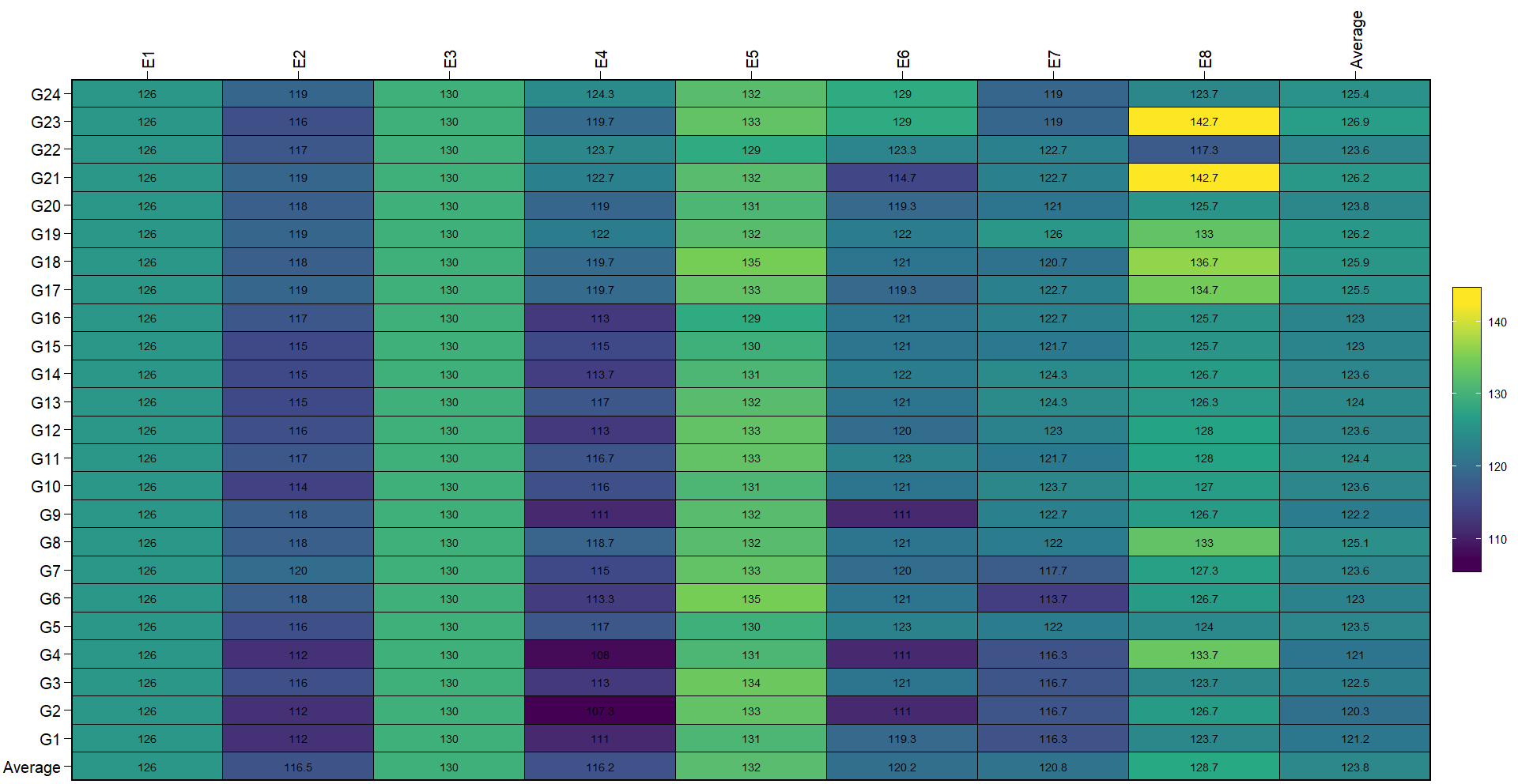 | 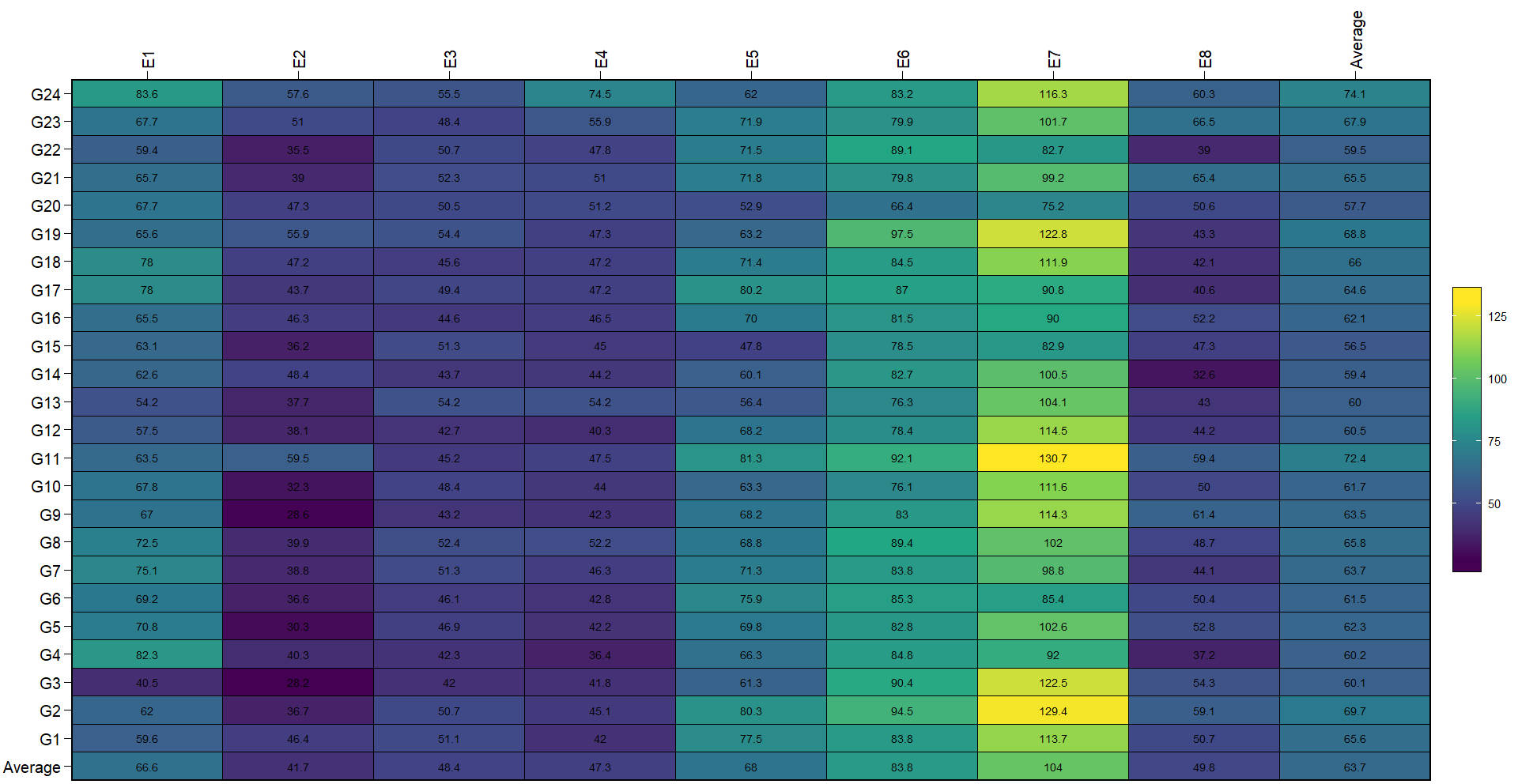 |
| **TSS** | **DB** | **TI** |
| 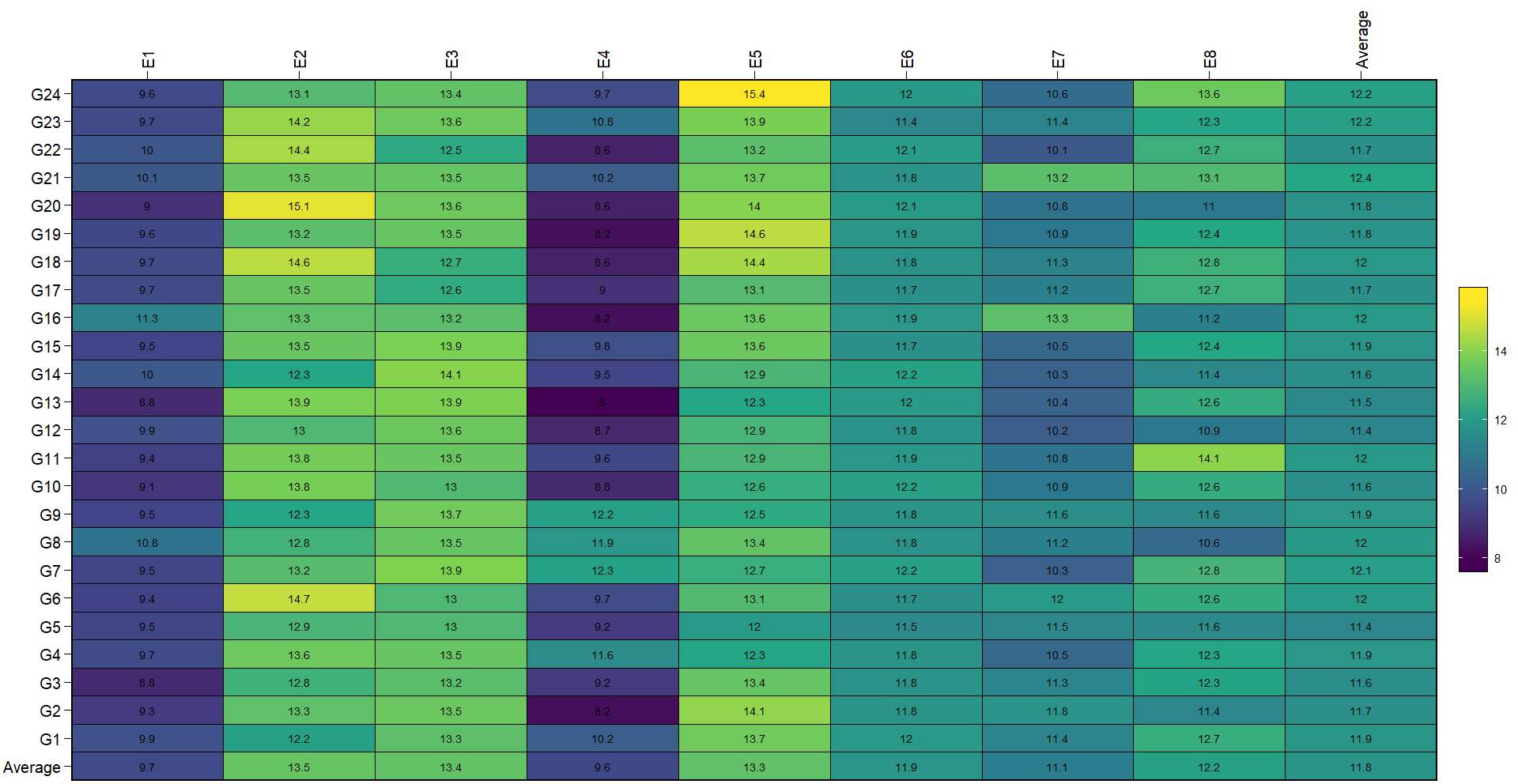 | 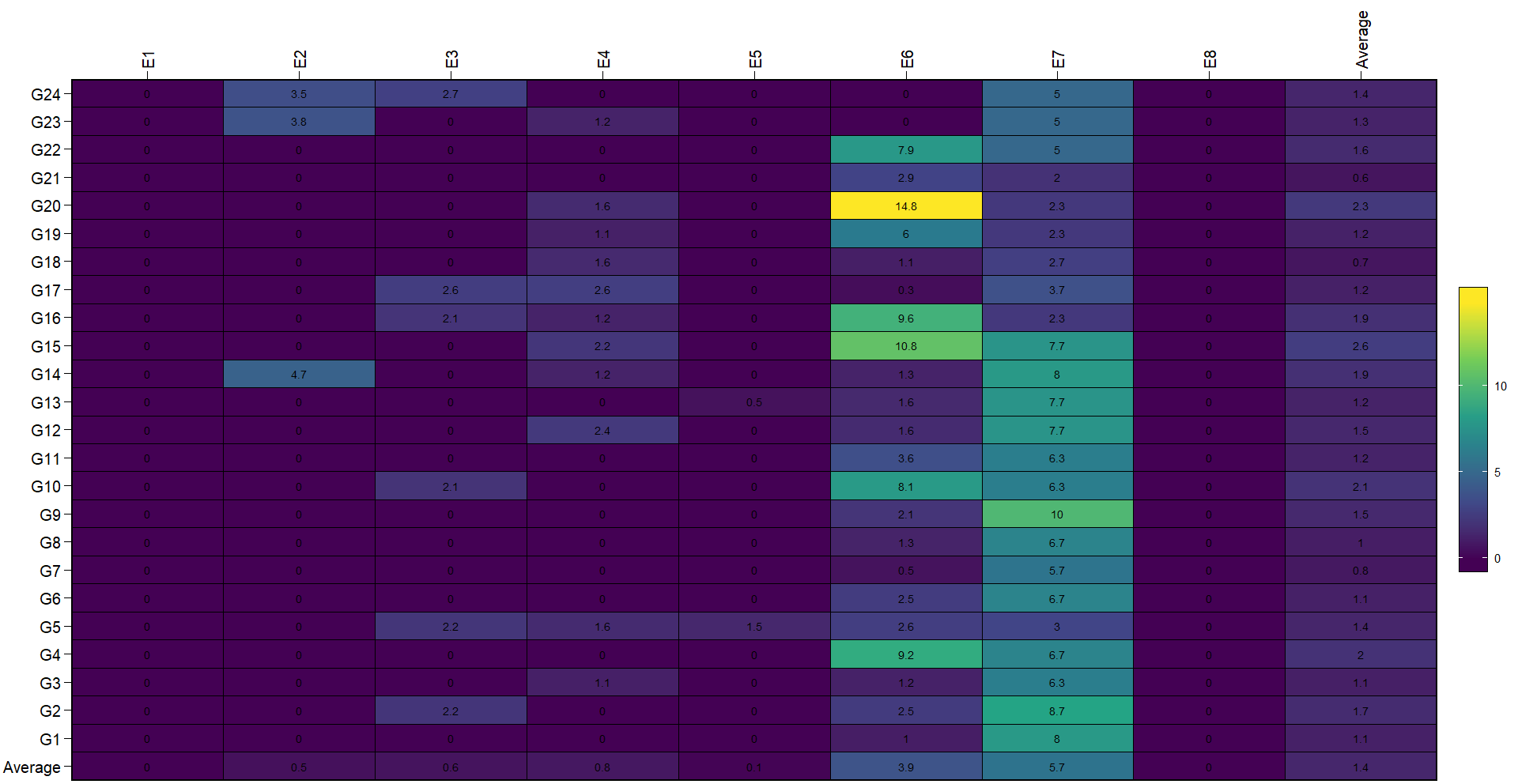 | 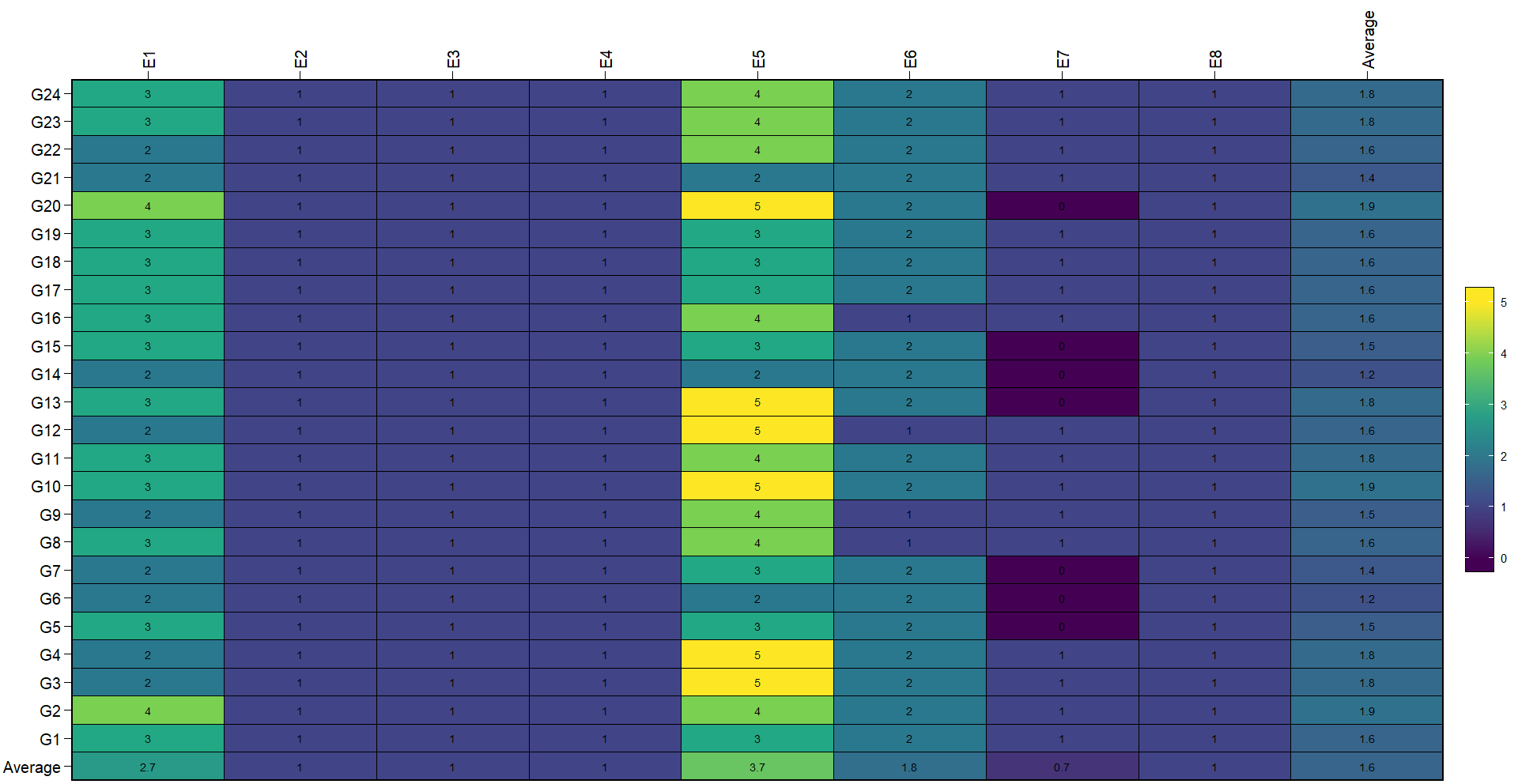 |

**Supplementary File 2.** Heatmap visualization of genotype performance across eight test environments: MY: marketable yield; DTH: days to harvest; ABW: average bulb weight; TSS: total soluble solids; DB: double bulb incidence; and TI: thrips incidence
